# Membrane Association of the PTEN Tumor Suppressor: Molecular Details of the Protein-Membrane Complex from SPR Binding Studies and Neutron Reflection

**DOI:** 10.1371/journal.pone.0032591

**Published:** 2012-04-10

**Authors:** Siddharth Shenoy, Prabhanshu Shekhar, Frank Heinrich, Marie-Claire Daou, Arne Gericke, Alonzo H. Ross, Mathias Lösche

**Affiliations:** 1 Physics Department, Carnegie Mellon University, Pittsburgh, Pennsylvania, United States of America; 2 Center for Neutron Research, National Institute of Standards and Technology, Gaithersburg, Maryland, United States of America; 3 Department of Biochemistry and Molecular Pharmacology, University of Massachusetts Medical School, Worcester, Massachusetts, United States of America; 4 Department of Chemistry and Biochemistry, Worcester Polytechnic Institute, Worcester, Massachusetts, United States of America; 5 Department of Biomedical Engineering, Carnegie Mellon University, Pittsburgh, Pennsylvania, United States of America; Massachusetts Institute of Technology, United States of America

## Abstract

The structure and function of the PTEN phosphatase is investigated by studying its membrane affinity and localization on in-plane fluid, thermally disordered synthetic membrane models. The membrane association of the protein depends strongly on membrane composition, where phosphatidylserine (PS) and phosphatidylinositol diphosphate (PI(4,5)P_2_) act pronouncedly synergistic in pulling the enzyme to the membrane surface. The equilibrium dissociation constants for the binding of wild type (*wt*) PTEN to PS and PI(4,5)P_2_ were determined to be *K_d_*∼12 µM and 0.4 µM, respectively, and *K_d_*∼50 nM if both lipids are present. Membrane affinities depend critically on membrane fluidity, which suggests multiple binding sites on the protein for PI(4,5)P_2_. The PTEN mutations C124S and H93R show binding affinities that deviate strongly from those measured for the *wt* protein. Both mutants bind PS more strongly than *wt* PTEN. While C124S PTEN has at least the same affinity to PI(4,5)P_2_ and an increased apparent affinity to PI(3,4,5)P_3_, due to its lack of catalytic activity, H93R PTEN shows a decreased affinity to PI(4,5)P_2_ and no synergy in its binding with PS and PI(4,5)P_2_. Neutron reflection measurements show that the PTEN phosphatase “scoots" along the membrane surface (penetration <5 Å) but binds the membrane tightly with its two major domains, the C2 and phosphatase domains, as suggested by the crystal structure. The regulatory C-terminal tail is most likely displaced from the membrane and organized on the far side of the protein, ∼60 Å away from the bilayer surface, in a rather compact structure. The combination of binding studies and neutron reflection allows us to distinguish between PTEN mutant proteins and ultimately may identify the structural features required for membrane binding and activation of PTEN.

## Introduction

Lipid-mediated cell signaling with phosphatidylinositol phosphates (PIPs) results in exquisite spatio-temporal control of vital cell functions. The rich functionality of the PIP headgroup with multiple phosphorylation sites on the inositol ring provides for selective interactions with a broad range of target proteins. By interconverting different PIP species through phosphorylation or dephosphorylation, kinases and phosphatases define the membrane distributions of the various PIP species temporally and spatially. As an interfacial phosphatase, phosphatase and tensin homologue deleted on chromosome 10 (PTEN) hydrolyses membrane-bound PI(3,4,5)P_3_ with high specificity for the 3-position of the inositol ring to produce PI(4,5)P_2_ [1]. Understanding the structural basis for PTEN regulation is critical because it plays an important role in many aspects of biology. PTEN is highly conserved in sequence, and we have insights into PTEN's functions for many species. In fission yeast, *Schizosaccharomyces pombe*, a PTEN analogue hydrolyzes PI(3,4,5)P_3_ and regulates vacuole morphology [2]. In the plant *Arabidopsis*, PTEN is expressed in pollen grains and is required for pollen maturation and survival [3]. PTEN has an asymmetric distribution in the slime mold *Dictyostelium discoideum*, resulting in accumulation of PI(3,4,5)P_3_ at the leading edge of migrating cells [4]. PTEN in honeybees plays a role in nutrient sensing and, thereby, queen-worker differentiation [5]. DAF-18 is a PTEN homolog in *Caenorhabditis elegans* worms that regulates aging [6]. In mice, loss of PTEN is embryonic lethal. PTEN affects many organs, but its roles in the nervous and immune systems have been particularly well studied [7]–[9].

With its function as a phosphatase, PTEN is the major antagonist to PI-3-kinase (PI3K), limiting basal levels of PI(3,4,5)P_3_ in the inner leaflet of the plasma membrane. Because elevated PI(3,4,5)P_3_ levels lead to unconditional cell survival and growth, PTEN inactivation is associated with multiple disease states, including many types of cancer [10]. In fact, PTEN is the second most commonly mutated protein in sporadic human tumors [11], and its activity as a tumor suppressor is essential [12]. More recently, roles of PTEN in human behavior have been identified. For example, the protein suppresses responses to drugs of abuse [13], and PTEN mutations were observed in autistic patients with macrocephaly [14]–[20]. Despite its importance, our current knowledge of the regulation of PTEN-PIP interactions is largely indirectly based on the analysis of cancer mutations [21]. Clearly, an assessment of the molecular origin of PTEN's membrane association and phosphatase activity will benefit our understanding of key processes in organisms from yeast to humans.

From the N-terminus to the C-terminus, PTEN consists of a PI(4,5)P_2_-binding module (PBM), the predominantly α-helical phosphatase domain (PD), a C2-domain dominated by β-sheet, and a ∼50 amino acid (AA) C-terminal tail that includes two PEST motifs and a PDZ motif. Plasma membrane binding of PTEN is partially electrostatically driven, with phosphatidylserine (PS) and PI(4,5)P_2_ being the main anionic phospholipids in the cytosolic leaflet that interact with PTEN [22]. The N-terminus forms the PBM and may thus initiate the binding process [21]. By binding to PS, the C2 domain helps target the protein to the bilayer where it hydrolyzes its substrate PI(3,4,5)P_3_. Membrane specificity is provided by selective binding of the PBM to PI(4,5)P_2_, targeting the protein to the plasma membrane, which is particularly rich in PI(4,5)P_2_ compared with other intracellular membranes. On the other hand, phosphorylation of the C-terminal tail abrogates membrane binding of the protein [23], thus providing another level of control of PTEN [21].

The structural arrangement and localization of PTEN at the membrane are currently poorly understood. The only crystal structure of PTEN available [24] shows that the C2 domain and the PD stabilize each other through interdomain contacts, but this structural model was developed with a truncated protein where parts of the N-terminus (AA residues 1–6), an interconnecting loop on the C2 domain (AAs 286–309) and the C-terminal tail (AAs 354–403) were deleted to allow crystallization. In addition, peptide segments adjacent to these deletions are not defined in the crystal structure. Hence, we do not have a structural basis to ask: how does the complex network of interactions between lipid species and protein binding domains control PTEN's enzymatic activity at the membrane surface? Which structural aspects are characteristic of PTEN membrane association, and how is membrane association altered by mutations at the protein-membrane interface? How do such structural alterations translate into changes in the membrane affinity of PTEN?

In this work, we develop methodologies to approach such questions. The key challenge here is to characterize the system—protein and membrane—at high resolution while the bilayer is in a thermally disordered, fluid state. We have recently demonstrated that this can be indeed achieved by analyzing neutron reflection (NR) [25] from substrate-supported, in-plane fluid, lipid membranes [26]. The membrane model systems used in these studies, sparsely-tethered bilayer lipid membranes (stBLMs), have been thoroughly studied in terms of their structural [26], functional [27] and dynamic properties [28]. In such studies, NR provides the one-dimensional (1D) neutron scattering length density (nSLD) profiles of the interfacial structures [29], *i.e.*, projections of the material distribution along the surface normal. However, by accounting for molecular volumes and properties, chemical connectivities and the internal structure of the protein, the “missing dimensions" can be filled in by computer models [30]–[32]. In such cases, one can resolve with high resolution the spatial association [33] and orientation [34] of a protein on the bilayer. In the case of PTEN, the situation is more complex, because ∼20% of the AAs of the *wt* protein are either omitted or not resolved in the crystal structure. In addition, a recent IR investigation showed that the protein undergoes conformational changes upon membrane binding [35]. Despite these complications, we describe here the first steps to resolving the structure of a carefully designed PTEN/membrane model system.

## Materials and Methods

### [36] Preparation of PTEN Protein

Human PTEN with a six-histidine tag at the C-terminus was expressed in Escherichia coli BL21 (DE3) bacteria as described [35]. We produced wild-type (*wt*), an enzymatically disabled mutant (C124S PTEN) [37], an autism-related H→R mutant (H93R PTEN) [38] and a truncated PTEN whose crystal structure was reported [24]. The plasmid of the truncated PTEN was provided by Dr. N. R. Leslie (University of Dundee). PTEN proteins were purified with a HisTrap HP kit from GE Healthcare, a Superdex 200 column and a MonoQ anion-exchange column. For SPR or NR, the protein was centrifuged at 13,200 rpm for 10 min., dialyzed in 100 mM NaCl, 10 mM HEPES, 1 mM EDTA, 1 mM β-mercaptoethanol (βME), pH 7.0 (buffer **1**) overnight using a Slide-A-Lyzer dialysis cassette (Thermo Scientific, mod. 66370), centrifuged again for 10 min., and the supernatant used. Unless otherwise stated, reagents were of p.a. grade and obtained from Sigma-Aldrich.

### Preparation of stBLMs

3⋅1⋅1 mm glass slides from Fisher Scientific (for SPR) and 3, 380 µm thick, [100]-cut Silicon wafers (Silicon Quest International; for NR) were cleaned first in 5 vol% Hellmanex (Hellma GmbH) and then Nochromix (85 g per 2.5 L of 18 M H_2_SO_4_; Godax Laboratories), followed by excessive rinsing with ultrapure water (Millipore) and absolute EtOH (Pharmco-Aaper) and drying in an N_2_ gas stream. They were coated with Cr (∼20 Å) and Au (∼150 Å and ∼450 Å for NR and SPR, respectively) by sputtering in a high-energy magnetron (ATC Orion; AJA International) at 0.15 µTorr. Some of the samples produced for NR experiments incorporated an iron-nickel (“permalloy") bonding layer instead of Cr. After the sputtering, x-ray reflectometry (JJ X-ray/Bruker AXS) showed a typical RMS surface roughness of ∼5 Å of the gold film. After sputtering, the gold-coated Si wafers were immediately transferred into a 3∶7 (mol∶mol) 0.2 mM (total concentration) ethanolic solution of *Z* 20-(*Z* octadec-9-enyloxy)-3,6,9,12,15,18,22-heptaoxatetracont-31-ene-1-thiol (HC18; structure, see figure in Information S1) and βME to form a self-assembled monolayer (SAM). HC18 was a kind gift from Dr. D. J. Vanderah (Institute for Bioscience and Biotechnology Research, IBBR, Rockville, MD), synthesized similarly to related compounds described earlier [26], [39]. stBLMs were completed by precipitation of phospholipids on the preassembled SAM by rapid solvent exchange (RSE) [26], [40]. Dipalmitoyl- (DP-)PI(4,5)P_2_ and DPPI(3,4,5)P_3_ were from Cayman Chemical. Brain-derived PI(4,5)P_2_ and all other phospholipids and cholesterol were from Avanti Polar Lipids. At *T* = 45°C, ethanolic solutions of lipid mixtures (∼5 mg/mL) of the appropriate compositions were used to incubate the SAM-covered substrates, followed by a rapid replacement with aqueous buffer **1**. To avoid the formation of incomplete bilayers, phospholipid mixtures that contained ≥20 mol% of anionic lipids were supplemented with 5 mol% cholesterol. For compositions that contain ≤40 mol% total anionic lipids, this procedure leads to the formation of complete bilayers in the stBLMs, as observed with electrochemical impedance spectroscopy (EIS) and NR (Shenoy, Heinrich and Lösche, unpublished results).

### Surface Plasmon Resonance (SPR) and Electrochemical Impedance Spectroscopy (EIS)

Glass slides with ∼450 Å of gold were coupled to a prism in a custom-built SPR instrument from SPR Biosystems (Germantown, MD) in the Kretschmann configuration. A superluminescent LED (EXS7510, Exalos AG, Switzerland) excited surface plasmons in the gold film at λ = 763.8 nm. The LED emission was focused on the sample by means of a hemicylindrical prism, such that a range of incident angles were covered on the sample, reflected, and collected on a position-sensitive CCD detector. The illuminated sample area was 10 mm×100 µm with the long side perpendicular to light propagation. The reflected light was collected on a CCD (Hamamatsu C10990) with 250 lines of 1024 pixels at 25 µm resolution. In the standard, single channel measurement mode, all 250 lines were binned into one line of 1024 pixels. The system had a time resolution of 0.1 s with a sensitivity of 5×10^−7^ reflectivity units (RU) or better. The dynamic range in refractive index was *n* = 1.33 to 1.41. A temperature controller (Wavelength Electronics LFI-3751) was used with a range from ambient to 50°C with 0.005°C resolution. The sample cell was composed of a Teflon cylinder of 6 mm inner diameter with a volume of ∼1 mL. A modified IKA (Wilmington, NC) RW11 overhead stirrer was used following injection of protein into the sample cell.

Simultaneously with the SPR measurement, the instrument allowed EIS measurements using a Solartron 1287A potentiostat and 1260 frequency response analyzer if the stirrer was not in use. In standard data acquisition mode, the position, *R*, of the reflection minimum as a function of incident angle within the convergent incident beam was determined at 0.2 s intervals. The EIS response was measured with a saturated silver-silver chloride (Ag|AgCl|NaCl(aq,sat)) microelectrode (Microelectrodes, mod. M-401F) with the auxiliary electrode consisting of a 0.25 mm diameter Pt wire (99.99% purity, Aldrich) coiled around the barrel of the reference electrode and the gold film used as the work electrode. Results were fitted to equivalent circuit models [26], [27] using ZView (Scribner Associates, Southern Pines, NC).

In a standard equilibrium binding assay in SPR, a given concentration of protein, *c_p_*, was added to a stable bilayer. The increase in the SPR signal *R*, due to an increase in refractive index at the bilayer/buffer interface was attributed to protein binding at the bilayer. Once *R* ceased to change, equilibrium was assumed to have been attained at *R_eq_*. A higher concentration of protein was then added to repeat the adsorption process. The change of *R_eq_* as a function of *c_p_* was fitted to [41]

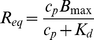
(1)where *K_d_* is the equilibrium dissociation constant and *B*_max_ is the saturation of the SPR response at *c*→∞. The calibration of the instrument is described in Information S1.

### Neutron Reflection

NR measurements were performed on the Advanced Neutron Diffractometer/Reflectometer (AND/R) [42] or the NG1 reflectometer at the NIST Center for Neutron Research (NCNR). The sample was assembled in a dedicated flow-through sample cell, such that the fluid adlayer in the sample that baths the stBLM was continuously exchanged by means of a microfluidic pump (MP5 with MP-X controller, Bartels Mikrotechnik GmbH). The resilience of the stBLMs permitted the NR characterization of the membrane at various solvent contrasts with the same physical sample. For contrast variation, exchanges of the buffer phase bathing the stBLM were performed *in situ* on the instrument, so that the neutron spectra were taken on exactly the same footprints on the wafers. This ensured that the inorganic substrates, in particular the SiO_x_/Cr/Au or SiO_x_/Fe-Ni/Au surface layers that dominate the interference patterns in the data, contributed identically to subsequent measurements. For each contrast, the reflectivity was measured at *T* = 25°C over a momentum transfer, *q_z_*, range of 0.008 to 0.25 Å^−1^, taking typically 6 h to obtain sufficient statistics. Protein adsorption to a preformed stBLM was accelerated by continuously pumping protein solution through the sample before starting the measurement scan.

NR data were analyzed using the *ga_refl* software package developed at NCNR (see Appendix A in ref. [43]). We adapted a recently developed model which describes the distribution of the stBLM components in terms of thermally broadened distributions of molecular fragments along the surface normal [44]. We refer to this parameterization as the *continuous distribution* (CD) approach to data modeling. Catmull-Rom splines [45] were used to parameterize the distribution of the protein normal to the bilayer with a constant protein nSLD derived from the PTEN crystal structure. The spline profiles were constrained to be single-peaked. The structural relation of the protein profile with the lipid bilayer was established by letting a parameter that describes position of the support point of the spline closest to the membrane surface adjust to a position within or outside of the lipid headgroup. The resulting compound nSLD profile, ρ*_n_*(*z*) still depends on the isotopic solvent contrast used in a particular NR scan. Multiple data sets from distinct isotopic contrasts with and without protein were all simultaneously fitted, thus generating a set of nSLD profiles consistent in terms of the underlying molecular structure [30], [31]. The quality of the fit is quantified as the normalized χ^2^ deviation between the model and the experimental data. To determine the variability of the resulting nSLD profiles within the experimental uncertainties, to quantify confidence intervals and to track correlations between the fit parameters, a Monte-Carlo resampling procedure [39] was implemented in *ga_refl*.

For a comparison of experimental nSLD profiles with the putative nSLD distribution of the truncated PTEN, we aligned the crystal structure with the membrane as proposed by Lee and coworkers [24]. As in earlier work [33], we then determined the nSLD in 0.5 Å thick slices parallel to the interface according to their atomic contents and solvent-excluded partial protein volumes measured using Connolly's method [46] with a probe radius of 1.4 Å. The resulting nSLD distribution was subject to Gaussian smoothing with the σ value determined from the modeling of the experimental nSLD profile, because this parameter is essentially determined by the roughness of the gold surface. Finally, the resulting PTEN profile was scaled to the maximum of the protein contribution to the nSLD profile and its position adjusted along *z* for maximum overlap with the experimental protein distribution.

## Results

In this work, we seek a solid understanding of the molecular factors that determine PTEN membrane binding. This was accomplished with SPR measurements on stBLMs of various binary and ternary compositions for which DOPC was the invariable majority component that quantified affinities and saturating protein concentrations for *wt* PTEN and various mutants. We also report NR measurements on the association of *wt* PTEN mutant proteins to stBLMs, which provide direct evidence of the protein structure on an in-plane fluid, thermally disordered membrane.

The most abundant anionic component of the cytosolic leaflet of mammalian plasma membranes, where PTEN exerts its phosphatase activity, is phosphatidylserine (PS). PI(4,5)P_2_ and phosphatidic acid (PA) contribute to electrostatic interactions to a lesser extent. The relevant concentrations of these lipids in the plasma membrane are approx. 25% (PS), <5% (PA) and ∼1% (PI(4,5)P_2_). Finally, PTEN's substrate, PI(3,4,5)P_3_, contributes only minimally to membrane charge, even though it is highly charged, as it is present only in minuscule concentrations [47]. In distinction, the plasma membrane is the major cellular repository of PI(4,5)P_2_, with a global concentration estimated to be ∼1%.

SPR experiments on stBLMs precisely determine protein binding affinities and are invaluable as precursor experiments, conveniently optimizing sample preparation for the scattering experiments. We prepared stBLMs of various compositions of DOPC, DOPS and PI(4,5)P_2_. For membranes with greater than 10 mol% anionic lipids, we routinely added 5 mol% of cholesterol (chol) to the lipid mixture in RSE preparations because bilayers without chol were less complete, typically 95% coverage of the surface by the membrane. While chol may affect the morphology of PI(4,5)P_2_-containing membranes at higher concentrations, such low concentrations of cholesterol apparently do not impact the lateral distribution of PI(4,5)P_2_ (Gericke and Jiang, unpublished data). To study PTEN interactions with PS, we compared *wt* PTEN with H93R PTEN, whose affinity to PS is significantly enhanced [38]. The association of these proteins was further studied on stBLMs that contained PI(4,5)P_2_ as the only anionic lipid and to membranes with both PS and PI(4,5)P_2_. We also studied the binding of C124S PTEN to PIP_3_-containing stBLMs to quantify interaction with its specific substrate. By investigating these point mutants, one may also obtain information on the plasticity of the protein. Furthermore, we investigated the truncated PTEN variant used in the crystallization study [24], which will allow us to use the crystal structure as a starting point for our NR-determined structures.

### PTEN Membrane Affinities

**Figure 1:**
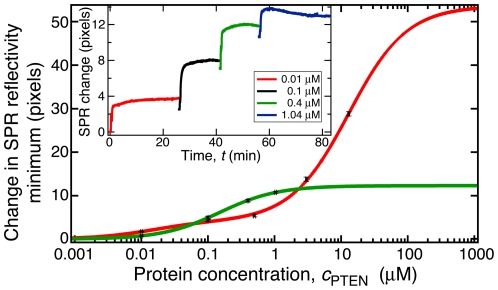
Quantification of PTEN Binding to stBLMs by Surface Plasmon Resonance. Exemplary SPR results showing a raw data set (binding of *wt* PTEN to an stBLM composed of DOPC and ∼2 mol% PI(4,5)P_2_; inset) and two binding isotherms (main panel). The green isotherms shows a fit to the data in the inset, which are well described by assuming a simple Langmuir adsorption (Eq. 1) with *K_d_*∼300 nM. In distinction, the isotherm shown in red (binding of *wt* PTEN to an stBLM composed of DOPC, ∼28 mol% DOPS and ∼2 mol% PI(4,5)P_2_ with chol) shows bimodal binding behavior of the protein to the two anionic components in the bilayer in which one component has a *K_d_* of ∼40 nM and the second has a *K_d_* of ∼10 µM. While the first of these values is smaller than the *K_d_* of PTEN binding to PI(4,5)P_2_ alone (see cross-over of the two curves in the low-concentration regime), the second *K_d_* coincides approximately with that measured for *wt* PTEN binding to stBLMs that contain only DOPS as a single anionic component. A conversion of the raw data into equivalent protein mass per unit area is given in Information S1.

Earlier work showed that the binding affinity of PTEN to DOPC bilayers is low (>500 µM) [35]. Here, we performed SPR in a custom-designed sample environment that permits the preparation of stBLMs by RSE *in situ*, as well as the simultaneous measurement of SPR and EIS. This enables characterization of stBLMs quality before the injection of protein into the sample chamber. Protein studies were only conducted with bilayers that were >99% complete. Figure 1 shows an SPR binding curve of *wt* PTEN to an stBLM composed of DOPC∶DOPS∶PIP(4,5)P_2_ = 68.8∶28.6∶2.6 (+chol). This binding curve shows two components with distinct affinities, *K_d_* ^(1)^∼0.04 µM and *K_d_* ^(2)^>10 µM. The solid line is the best fit to the data. Similar experiments with DOPC∶DOPS only or DOPC∶PIP(4,5)P_2_ only showed always single-component adsorption processes.

Two pieces of information are gleaned from such data. (1) The equilibrium binding constant *K_d_* quantifies the affinity of the protein to the membrane and is determined as the midpoint concentration of an adsorption process. It can be determined with confidence if the highest protein concentration (*c_p_*) sampled is well above the nominal value of *K_d_*, where the binding curve reaches half-saturation. In cases where the *c_p_* doesn't reach the midpoint, for example because of protein aggregation at high *c_p_*, *K_d_* can still be estimated as described in Information S1. A range of *K_d_* values, which is consistent with the truncated data, is given as the result in such cases. (2) The maximum SPR shift, *B_max_* at *c_p_*→∞, is a measure of the amount of adsorbed material. Complementary studies of protein adsorption to stBLMs showed that densely packed monolayers of protein on the membrane surface, as quantified with NR, show values in the range *R_eq_* = 175–300 ng/cm^2^ on the custom-built SPR instrument used in this study, depending on the physical size—and therefore the monolayer thickness—of the adsorbed protein (Shenoy and Lösche, unpublished data). Note that *K_d_* and *B_max_* may be uncorrelated, *i.e.*, the affinity and the amount of adsorbed protein are distinct characteristics of an adsorption process. For example, we frequently observe that the absorption of PTEN to PIP(4,5)P_2_ has high affinity (*K_d_*∼0.1 µM) with low amounts of protein adsorbed (*B_max_*<60 ng/cm^2^), while large amounts of PTEN protein bind to PS with low affinity (*K_d_*≥10 µM; *B_max_*>175 ng/cm^2^). Tables 1 and 2 summarize the equilibrium binding constants of the three PTEN constructs to stBLMs of various compositions. Specific results are presented below.

**Table 1:** Quantification of *wt* PTEN Binding to stBLMs by SPR.

Composition	*K_d_* (µM)	*B_max_* (ng/cm^2^)
**PC∶PS (+chol)**		
70∶30	11.9±0.4	155±3
**PC∶PI(4,5)P_2_**		
99.3∶0.7	0.4±0.1	23±1
97.8∶2.2	0.4±0.1	26±1
96.3∶3.7	0.4±0.1	71±2
**DPPC∶DPPIP(4,5)P_2_**		
96.3∶3.7	1.9±0.3	73±2
**PC∶PS∶PI(4,5)P_2_ (+chol)**		

	Component a	Component b	Component a	Component b
69.7∶29∶1.3	0.04±0.01	>5	22±1	>175
68.8∶28.6∶2.6	0.04±0.01	>5	29±12	>175
**PC∶PI(3,4,5)P_3_**				
97.9∶2.1	2.4±0.2		145±23	
**PC∶PI(4,5)P_2_∶PI(3,4,5)P_3_**				
96.4∶1.8∶1.8	1.0±0.1		93±35	

Dissociation constants, *K_d_*, in µM for the binding, at room temperature, of *wt* PTEN to stBLMs prepared by RSE on SAMs composed of HC18∶βME 70∶30 from lipid solutions of the compositions shown. The lipid chain compositions are dioleoyl (DOPC, DOPS) and that of the natural brain extract for PI(4,5)P_2_ (mostly stearoyl-arachidonoyl), with the exception of the sample denoted as **DPPC∶DPPI(4,5)P_2_**, for which the chain composition on both stBLM components is dipalmitoyl. With the exception of the system denoted as **PC∶PS∶PI(4,5)P_2_ (+chol)** which was clearly bimodal, all experiments were evaluated as single component fits.

**Table 2:** Quantification of mutant PTEN Binding to stBLMs by SPR.

Composition	*K_d_* (µM)	*B_max_* (ng/cm^2^)
***C124S PTEN***		
**PC∶PS (+chol)**		
70∶30	2.9±0.3	273±17
**PC∶PI(4,5)P_2_**		
97.8∶2.2	0.32±0.03	99±17
**PC∶PI(3,4,5)P_3_**		
97.9∶2.1	0.12±0.03	191±17
**PC∶PI(4,5)P_2_∶PI(3,4,5)P_3_**		
98∶1.8∶0.2	0.26±0.01	127±2
98∶1∶1	0.13±0.01	152±1
98∶0.2∶1.8	0.12±0.01	218±2
***H93R PTEN***		
**PC∶PS (+chol)**		
70∶30	3.1±0.3	163±17
**PC∶PI(4,5)P_2_**		
97.8∶2.2	1.3±0.2	64±6
**PC∶PS∶PI(4,5)P_2_ (+chol)**		
68.8∶28.6∶2.6	0.7±0.1	191±23
***truncated PTEN***		
**PC∶PS (+chol)**		
70∶30	2.5 … 4.9	350 … 560
**PC∶PI(4,5)P_2_**		
97.8∶2.2	0.77±0.07	81±3
**PC∶PS∶PI(4,5)P_2_ (+chol)**		
68.8∶28.6∶2.6	0.9±0.2	325±35

Dissociation constants, *K_d_*, in µM for the binding, at room temperature, of *wt* PTEN to stBLMs prepared by RSE on SAMs composed of HC18∶βME 70∶30 from lipid solutions of the compositions shown. The lipid chain compositions are dioleoyl (DOPC, DOPS) and that of the natural brain extract for PI(4,5)P_2_ (mostly stearoyl-arachidonoyl). All experiments were evaluated as single component fits. The parameter ranges given for adsorption of the truncated PTEN PC∶PS stBLMs were estimated as described in Information S1.

#### PTEN binding to single anionic lipid components in stBLMs with PC

Of the PTEN variants tested, *wt* PTEN shows the weakest binding affinity, *K_d_*≈12 µM [48], to DOPC membranes with DOPS as the sole anionic lipid. On the other hand, the *B* values reached at high protein concentrations (*B_max_*≈155 ng/cm^2^) suggest that we will be able to extract structural information from NR measurements. Given the differences in methodology, we consider this result to be consistent with the value, *K_d_* = 22.0±0.5 µM, reported earlier for a similar lipid composition [35]. H93R PTEN has a 4-fold higher binding affinity to PS with *K_d_* = 3.1±0.3 µM, again in agreement with earlier measurements using different techniques [38]. This is interesting, as the point mutation is in the PD—distant from both the PBM and C2 domains that are thought to mediate membrane association. As with *wt* PTEN, we were unable to determine *K_d_* for the truncated PTEN because we could not use large enough protein concentrations, as the protein aggregated above *c_p_*∼3 µM. *K_d_* values between 2.5 and 4.9 µM are consistent with the partial binding isotherms that we obtained.

The four PTEN proteins included in this study have highly distinct binding characteristics. The H93R PTEN mutant shows the weakest binding affinity to membranes that contain PI(4,5)P_2_ as the sole anionic component (*K_d_* = 1.3±0.2 µM for an stBLM with 2.2 mol% PI(4,5)P_2_). Although *K_d_* is a factor of 3 smaller for PI(4,5)P_2_ than PS, *B_max_*, and therefore the amount of bound protein, is ∼3× larger for PS-containing membranes than for PI(4,5)P_2_-containing membranes. The truncated PTEN mutant shows a two-fold stronger affinity and *wt* PTEN shows a four-fold stronger affinity to PI(4,5)P_2_ with *K_d_* ^(trunc)^ = 0.77±0.07 µM and *K_d_* ^(wt)^ = 0.4±0.1 µM, see Tables 1 and 2. A comparison of the affinities of *wt* and mutant PTEN shows that the *wt* protein has by far the lowest affinity to PS alone. While its affinity to PI(4,5)P_2_ is similar to those of the mutant proteins, its affinity to (PS+PI(4,5)P_2_) surprisingly is more than an order of magnitude greater than that of any of the mutants for which this lipid composition was studied. This indicates a remarkable synergy in binding of *wt* PTEN to (PS+PI(4,5)P_2_), which the mutants apparently lack.

To investigate the stoichiometry of PI(4,5)P_2_ binding to PTEN, we also studied the binding of *wt* PTEN to DPPC+DPPI(4,5)P_2_. Because we used lipids with saturated dipalmitoyl chains, the bilayer is expected to be in the gel phase at room temperature (DPPC has a melting point, *T_m_*∼41°C). As a result, the diffusivity of PI(4,5)P_2_ within the bilayer is low and prevents active recruitment of multiple PI(4,5)P_2_ molecules by PTEN, for example by electrostatic attraction. We observe that the binding affinity is approximately five-fold less (*K_d_* = 1.9±0.3 µM) to a membrane with saturated lipids compared to that with unsaturated lipids with the same headgroup composition. The fact that the *K_d_* values are distinct demonstrates the importance of bilayer fluidity in the binding, and argues against a 1∶1 binding stoichiometry between PTEN and PI(4,5)P_2_ [49].

Catalytic activity requires PTEN binding to PI(3,4,5)P_3_, dephosphorylation and production of PI(4,5)P_2_. We investigated the binding of *wt* PTEN and catalytically dysfunctional C124S PTEN to stBLMs composed of PC and PI(3,4,5)P_3_. The affinity of *wt* PTEN is six-fold larger to membranes that contain PI(4,5)P_2_ than to those containing PI(3,4,5)P_3_ (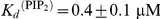; 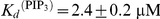). However, because the catalytically active *wt* protein converts its ligand in the process, these numbers are hard to compare. Similar measurements with the inactivated C124S mutant indeed show a slightly increased PTEN affinity to PI(3,4,5)P_3_-containing membranes (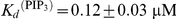) while the affinity to PI(4,5)P_2_-containing membranes is about the same for the wild type and the mutant (*K_d_* ^(C124S)^ = 0.32 µM *vs. K_d_* ^(*wt*)^ = 0.4 µM). This suggests that PI(3,4,5)P_3_ attracts the protein with a binding strength comparable to that of PI(4,5)P_2_, but following hydrolysis, the phosphatase active site binds PI(4,5)P_2_ less avidly, the PTEN is released more readily and hence, has a lower binding constant.

#### PTEN binding to dual anionic lipid components in stBLMs with PC

In mammalian cells, both PS and PI(4,5)P_2_ anionic lipids are implicated in PTEN's association with the lipid membrane. We prepared DOPC stBLMs containing 28.6 mol% PS and 2.6 mol% PI(4,5)P_2_. Both the H93R mutant and the truncated PTEN mutant show similar binding affinities with *K_d_* ^(H93R)^ = 0.7±0.1 µM and *K_d_* ^(trunc)^ = 0.9±0.2 µM. While *K_d_* for H93R binding to (PS+PI(4,5)P_2_) is comparable to that for binding to PI(4,5)P_2_ alone [50], the amount of bound protein is by a factor of ∼3 larger. In fact, it is larger than that for binding to PS alone, showing that the presence of both lipids maximizes the localization of PTEN on the membrane. For both H93R and the truncated PTEN mutant, the binding curves are well fitted by a single-component association model. However, the binding curves for *wt* PTEN show a strong bimodal signature, and while the higher *K_d_* is only approximate, due to a lack of high-concentration data, the lower one can be precisely determined to be *K_d_* = 0.04±0.01 µM. This is an order of magnitude stronger than association to PI(4,5)P_2_ alone—which in turn is nearly an order of magnitude stronger than PTEN association with PS—indicating a cooperative binding mechanism and once again reflecting the different roles of these anionic lipids in the membrane.

Finally, we also investigated *wt* PTEN and C124S PTEN binding to stBLMs containing both PI(4,5)P_2_ and PI(3,4,5)P_3_. In all cases, the binding curves were well described by a single-component model, presumably because the *K_d_* values for binding the individual lipid components are similar (Tables 1 and 2). For *wt* PTEN, the binding affinity (*K_d_* = 1.0±0.1 µM) is in between the values observed with each lipid independently. Note, however, that the same caution is needed in the interpretation of this result as with binding to PI(3,4,5)P_3_-bearing membranes, since catalytic conversion likely occurs during the experiments. For C124S PTEN, a continuous change of the single-component model *K_d_* values is observed between the respective values of 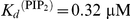 and 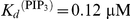 as the relative proportion of the two PIP components is tuned between PI(4,5)P_2_ and PI(3,4,5)P_3_ (with the overall PIP content in the membrane fixed at 2 mol%, see Table 2). This is consistent with non-competitive binding of the two PIP species, as expected, since the PBD targets PI(4,5)P_2_ and the catalytic site in the PD targets PI(3,4,5)P_3_.

#### Summary

In combination, the results presented above suggest distinct essential roles for all three anionic lipid components studied—PS, PI(4,5)P_2_, and PI(3,4,5)P_3_—in localizing PTEN to the membrane. At low PTEN concentrations, PI(4,5)P_2_ attracts PTEN to the bilayer by virtue of its rather high equilibrium binding constant. Whether PS merely provides a general background of electrostatic attraction or contributes an element of specific binding on its own is not clear; in any case, it leads to high protein coverage of the membrane surface at large (>1 µM) protein concentrations. Obviously, this suggests experimental conditions for preparing samples well suited for NR, *i.e.*, *B*_max_ >60 ng/cm^2^. Despite its strong interaction with PTEN, PI(3,4,5)P_3_ alone is insufficient to attract the phosphatase on its own, given its low concentration in the plasma membrane. Most interestingly, the cooperativity between anionic membrane components enhances binding of PTEN to membranes, as shown by the decrease of *K_d_* values measured for the same lipids in single and dual anionic component membranes. This effect is easily recognized by comparing PTEN binding to PI(4,5)P_2_ in membranes with and without PS. However, such cooperativity is not observed between PTEN binding to PI(4,5)P_2_ and PI(3,4,5)P_3_, or cannot be resolved because the individual *K_d_* values are too similar. Even if the affinity of PTEN to PI(3,4,5)P_3_ is slightly higher than to PI(4,5)P_2_, a threshold concentration of the latter may be required to attract the phosphatase to its target location, as indicated by the reduction of the affinity to membranes prepared from saturated lipids. It is therefore a pool of PI(4,5)P_2_, which distinguishes the composition of the inner plasma membrane from that of other cellular loci, that attracts PTEN to its interaction site.

### Neutron Reflection Results

**Figure 2:**
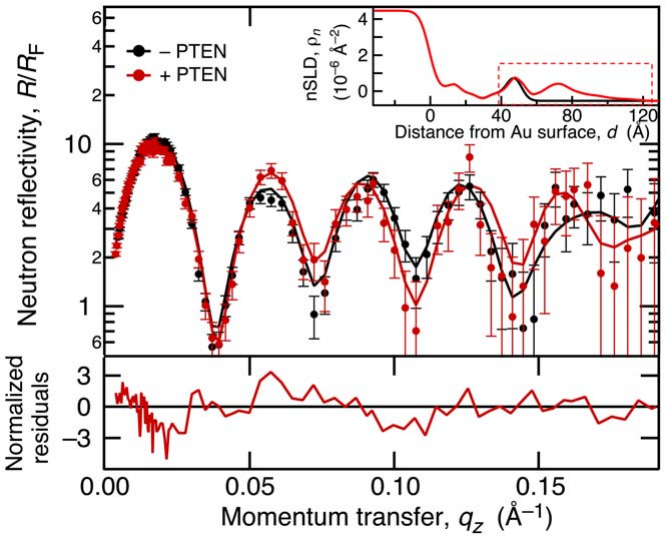
Quantification of PTEN Binding to stBLMs by Neutron Reflection. Exemplary NR data set showing the changes of the neutron reflection upon *wt* PTEN association with a preformed stBLM composed of DOPC∶DOPS 70∶30, the residuals which emphasize these changes (bottom), and the corresponding nSLD profiles (inset). The NR spectra (main panel) for the stBLM before (black) and after incubation with 20 µM *wt* PTEN (red) are normalized to the Fresnel reflectivity—*i.e.*, the reflectivity of a neat Si/buffer interface without interfacial roughness—in order to emphasize the interference patterns due to the interfacial structures. Changes of the spectra upon PTEN association with the membrane are shown as residuals, normalized to the magnitude of the experimental errors, at the bottom. Lines in the main panel show the computed NR of the nSLD profiles shown in the inset. Note that these profiles are derived by fitting multiple data sets simultaneously (neat stBLM and stBLM with PTEN, each measured at different isotopic buffer contrasts) by sharing model parameters as appropriate. A dashed box in the inset indicates the region of the distal lipid headgroups and associated PTEN protein, shown in close-up view in Fig. 4. The signal-to-noise in these measurements is comparable to that in similar studies on the incorporation of α-hemolysin into stBLMs [33] and the binding of the HIV-1 matrix protein to bilayer surfaces [34].

With experimental conditions for the binding of the PTEN mutants to stBLMs optimized by SPR, we began structural characterization of the membrane-associated proteins. NR experiments were performed on *wt* PTEN bound to (1) DOPC∶DOPS = 70∶30 (+chol) and (2) DOPC∶DOPS∶PI(4,5)P_2_ = 68.8∶28.6∶2.6 (+chol). In addition, we examined the binding of H93R to an stBLM of the same lipid composition as for sample (1). Figure 2 shows the NR data for sample (1). The main panel compares the Fresnel-normalized reflectivities of the as-prepared stBLM and the membrane after incubation with the protein in H_2_O-based buffer **1**. Error bars are based on neutron counting statistics and represent 68% confidence intervals. While the differences between the two NR spectra are small, the error-weighted residuals at the bottom of Fig. 2 show that they are significant. Similar spectra were measured for the as-prepared and protein-loaded membrane bathed in buffers based on D_2_O and on a mixture of H_2_O and D_2_O with a nSLD of ∼4×10^−6^ Å^−2^, denoted as “CM4". The inset displays nSLD profiles with and without protein for H_2_O-based buffer **1** derived from a CD model (see Methods section) that describes all data sets simultaneously. Differences occur exclusively in the region at and beyond the distal lipid headgroups (dashed box) 40–100 Å away from the gold surface (which defines *z* = 0). The full sets of data, co-refined nSLD profiles and corresponding sets of CD model parameters are provided in Information S1.

**Figure 3:**
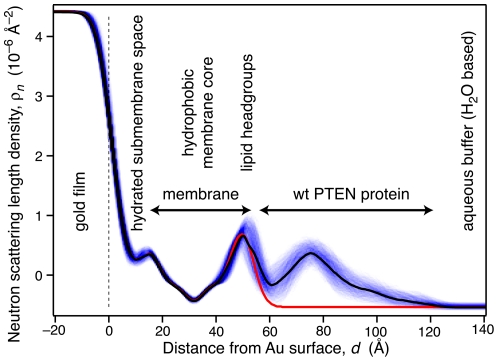
Neutron Scattering Length Density Profile of PTEN Protein Bound to a Lipid Bilayer in an stBLM. nSLD distribution of *wt* PTEN bound to a thermally disordered lipid membrane composed of DOPC with 30 mol% DOPS (+chol), as determined in a Continuous Distribution (CD) model. The sample is the same as that shown in Fig. 2. The red and black lines show those profiles for the as-prepared stBLM and the same sample after protein adsorption from a 20 µM PTEN solution, respectively, that derive from the most likely parameter set (Table 3). For the sample with PTEN protein, blue shaded contours visualize the bandwidth (1σ) of nSLD profiles that are also consistent with the experimental results, as determined by Monte-Carlo resampling of the data. The intensity of the shading corresponds to the probability of the model to be a true representation of the underlying structure.

The significance of the differences in scattering can also be explored in terms of the resulting nSLD profiles and their spread within experimental uncertainty. As described in detail in a recent publication [39], we resampled the experimental data within their neutron-flux limited errors with a Monte-Carlo algorithm to obtain a family of nSLD profiles (typically, 1000 iterations) that are consistent with the data given the error bars. As an example, Fig. 3 shows the resulting ensemble of nSLD profiles of the interfacial region that encompasses the stBLM and adsorbed PTEN protein for the same system as in Fig. 2. Darker colors indicate more likely values for the nSLD profile, while lighter coloration indicates values with a low likelihood. This depiction provides a visual assessment of the spread in the nSLD distribution that is consistent with the experimental result. In addition, black and red solid lines show the most likely nSLD profiles for the stBLM with and without adsorbed *wt* PTEN protein, respectively. (For clarity, the spread of nSLD profiles for the as-prepared stBLM is not shown.) The model parameters that give rise to the nSLD ensembles are narrowly distributed about their most likely values. The widths of these parameter distributions were used to determine their confidence limits [39], listed in Table 2 (and in more detail in Information S1).

**Figure 4:**
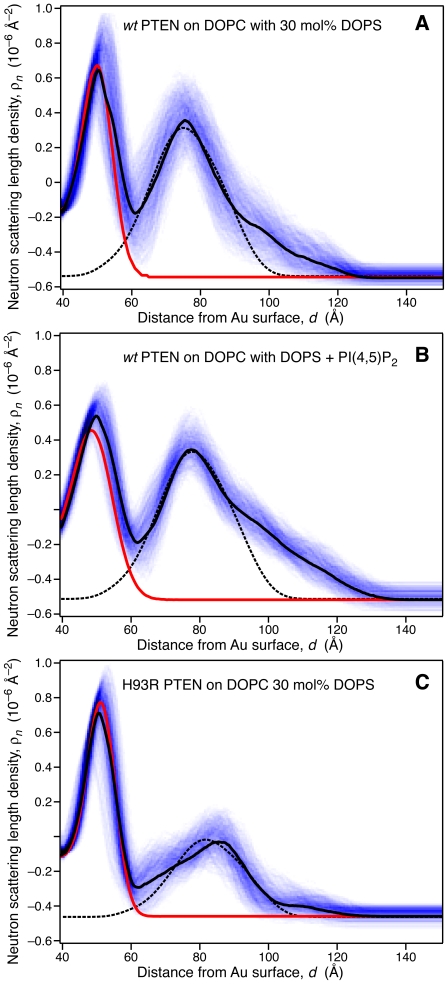
Comparison of nSLD profiles of PTEN Interfacially Bound to Lipid Bilayers and Their Correspondence with the Crystal Structure of the Truncated PTEN. The protein contributions (*d* = 60–120 Å from the Au surface) to the overall nSLD profile correspond to *wt* PTEN adsorbed to stBLMs (A) with 30 mol% DOPS (+chol), (B) with 28.6 mol% DOPS and 2.6 mol% PI(4,5)P_2_ (+chol), and to H93R PTEN adsorbed to an stBLM with 30 mol% DOPS (+chol). The peak near *d* = 50 Å originates from the lipid headgroups. The color coding is the same as in Fig. 3, and panel (A) is a close-up view of the results shown there. Dashes black lines represent the best-fit overlay of a scaled nSLD distribution calculated from the crystal structure of the truncated PTEN variant. Because different amounts of protein was adsorbed in the individual experiments, the ratios between the protein and lipid headgroup peaks varies from sample to sample.

Figure 4 displays magnified views of the same result for sample (1) (panel A) and the result for sample (2), in which the stBLM contained 2.6 mol% of PI(4,5)P_2_ in addition to PC, PS and chol. The coloration is the same as in Fig. 3. In addition, we show (dashed lines) a putative nSLD profile of the truncated PTEN protein derived from the crystal structure. In both cases, we observed only minimal penetration of the protein into the lipid headgroup region, indicating that PTEN interaction with the membrane is purely an interfacial phenomenon. A close inspection of the nSLD profiles shows that both protein structures at the interface are almost identical, and the region of the nSLD attributed to the protein which is proximal to the membrane is in both cases very well approximated by the putative nSLD distribution derived from the PTEN crystal structure. (The ratio of the peak nSLD values of lipid headgroups and protein is slightly different, as it depends on the amount of adsorbed protein, and hence on details of the experimental procedure.) On the distal end of the protein nSLD distribution, at *z* = 100–120 Å from the gold surface, we observe some extra density. It is tempting to speculate that the C-terminal tail of the *wt* protein, truncated in the crystal structure, contributes the scattering in that region of the profile.

## Discussion

This study serves two major objectives. (1) Map out the contributions of various anionic lipids of the inner plasma membrane to PTEN membrane binding and (2) establish experimental conditions for structural studies of the PTEN/membrane complex in the physiologically relevant, disordered state of lipid bilayers. The stBLM system is well suited both for precise binding affinity determinations via SPR and NR measurements of thermally disordered interfacial structures.

PTEN is a cytosolic protein that associates with membranes only if specific conditions are met [21]. As a result, only a small fraction of the pool of cellular PTEN is bound to the membrane at any time, and that pool is exquisitely controlled with respect to temporal and spatial distributions. PTEN's targeting and activation requires binding to a distinct ensemble of lipids, and the level of binding is a major determinant of its activity. Our well-controlled model membrane systems allow comparison of binding of *wt* protein with that of mutant proteins to gain insights into the role of membrane composition in PTEN regulation. The methodology used to quantify PTEN binding in this work offers significant improvements that derive from the stBLM sample format. These improvements include continuous, virtually defect-free single bilayer interfaces that permit quantitative measurements without the need for reference surfaces; suppression of unspecific protein binding; long-term stability; and the compatibility of the sample format with multiple complementary characterization techniques such as electrochemical impedance spectroscopy, fluorescence correlation, fluorescence microscopy, and reflectivity measurements, harnessed here to obtain a first glimpse on structure.

### Improvements in Quantitative Analysis of PTEN Membrane Binding

For SPR, carboxymethylated dextran chips are the conventional method to characterize protein binding to membranes and have been used to determine PTEN binding constants [22]. However, *K_d_* values obtained with that technology were by a factor of ∼1,000 smaller than those determined by other methods [35]. Narayan and Lemmon offer an explanation in their discussion of SPR analysis of phosphoinositide binding domains [51]. *K_d_* values are often obtained through a kinetic analysis from the calculation of *k_on_*/*k_off_* ratios. Such a kinetic analysis of SPR data yielded apparent affinities which were 10–1000-fold higher than those obtained by other equilibrium-based methods (*e.g.*, titration calorimetry or sedimentation analysis) [51]. While the reasons for these discrepancies remain unknown, unspecific protein binding to the carboxymethylated dextran chip, due to incomplete lipid coverage, might be at least part of the problem. In contrast, an SPR saturation analysis of steady state values reached for each protein concentration yielded equilibrium binding values that match the data obtained by alternative methods (for a detailed discussion see ref. [51]). In this work, we adopt a similar approach for the evaluation of *K_d_* values, however, instead of using a carboxymethylated dextran chip with an unknown lipid coverage, we used stBLMs that cover the chip surface with virtually defect-free, single bilayers, as determined by NR [26], [39]. For the SPR analysis of phosphoinositide binding domains, including PTEN, stBLMs offer significant advantages: First, the combination of SPR and EIS allows us to monitor the quality of the lipid bilayer *in situ*. Specifically, EIS has the sensitivity of determining the specific resistivity of a bilayer over many orders of magnitude and showed that stBLMs are virtually defect-free [26]. Second, because bilayers within stBLMs are complete (>99%, as shown by EIS and NR [26], [33], [39]) and defect-free, non-specific binding to the chip surface can be ruled out, and therefore SPR measurements do not need to be referenced to a phosphatidylcholine covered chip surface. It was noted that protein binding to PC cannot be determined using the carboxymethylated dextran chips because of the lack of an appropriate reference for such a measurement [51]. In our case, it is possible to obtain binding data for any lipid system, including PC. Third, the long-term stability of lipid-covered carboxymethylated dextran chips is an issue [51], which limits their use to less than 8 hours. In contrast, stBLMs have been shown to be stable for months [52]. An additional advantage that stBLMs offer in comparison to carboxymethylated dextran chips is the cross-platform utility of the model system. stBLMs can be used to characterize PTEN/membrane interactions using fluorescence techniques (e.g., imaging, FCS or single molecule measurements) or to study membrane structure by NR, as described in this paper.

### PTEN Affinities to Membranes of Different Lipid Compositions

It was recently shown that PTEN requires a distinct lipid environment for membrane association and activation. PTEN binds much more strongly to bilayers containing PS and PI(4,5)P_2_ than to bilayers that contain only one of these components [35]. From these data, we argued that the binding of PTEN to PI(4,5)P_2_ and PS is synergistic rather than competitive. The highly quantitative data presented here dramatically underscore this point (see Tables 1 and 2). For example, we find for *wt* PTEN that the affinity of the protein to bilayers with both lipids is an order of magnitude larger than the affinity to PI(4,5)P_2_ alone, and more than 2 orders of magnitude larger than to PS alone. Finally, this very high affinity binding was not observed for the H93R and truncated PTEN proteins, suggesting that this synergy requires multiple PTEN domains.

We also assessed the binding of PTEN to PI(3,4,5)P_3_-containing membrane. *wt* PTEN *apparently* shows weaker binding to PI(3,4,5)P_3_-bearing membranes than to PI(4,5)P_2_, both with the same PC bilayer background. Surprisingly, the binding is slightly lower for the ternary (PC+PI(4,5)P_2_+PI(3,4,5)P_3_) system than for the binary (PC+PI(4,5)P_2_) system. We can only speculate that structural adjustments imposed by PI(3,4,5)P_3_ on the protein that may prevent more effective binding. It is also likely that some of the PI(3,4,5)P_3_ substrate is converted to PI(4,5)P_2_ during the experiment, which prevents a clean analysis of the binding data. Because of this latter complication, we complemented the *wt* PTEN measurements with binding studies of C124S PTEN where the mutation, located in the catalytic binding pocket abolishes the protein's hydrolytic activity. Indeed, for C124S PTEN, we find that the binding to PI(4,5)P_2_ is similar to that of *wt* PTEN, *i.e.*, the mutation in the binding pocket does not affect the interaction of PI(4,5)P_2_ with the protein. However, the association of C124S PTEN with the PI(3,4,5)P_3_-containing stBLMs is significantly stronger than that of *wt* PTEN. There are two possible explanations. First, *wt* PTEN has an apparently weaker binding for PI(3,4,5)P_3_ because it converts it to PI(4,5)P_2_, which binds only weakly to the active site. Second, the C124S mutation may affect the PTEN-PI(3,4,5)P_3_ interactions at the active site. While an amino acid switch from Cys to Ser is neutral with respect to the space in the binding pocket to accommodate the substrate, there may be changes in the hydrogen bonding pattern to the ligand. The 3-phosphate of PI(3,4,5)P_3_ is close to the Ser side chain in the bound state, potentially allowing for the formation of a hydrogen bond that would increase binding strength to the PI(3,4,5)P_3_ headgroup. PI(4,5)P_2_ lacks the phosphate group in the 3-position, which explains why its binding is not affected by the mutation. It is also interesting that the binding of *wt* PTEN to PI(3,4,5)P_3_-containing membranes is significantly weaker than its binding to PI(4,5)P_2_. Binding studies with peptides derived from PTEN's N-terminus, which contains a PI(4,5)P_2_ binding site, showed that PI(3,4,5)P_3_ binding to PTEN's N-terminal end is minimal [35], suggesting that the observed PI(3,4,5)P_3_/PTEN interaction is largely at the active site. However, this binding is weaker than PTEN binding to PI(4,5)P_2_, even though the concentrations of the anionic lipids were the same in these experiments. We reported previously that the interaction with PI(4,5)P_2_ leads to an allosteric activation of PTEN [35], [53] that is not observed in the presence of PI(3,4,5)P_3_. Taken together, these observations suggest that PI(4,5)P_2_ binds to the N-terminal end of the protein, which augments the affinity of the protein to the membrane, possibly by locking in the bound state by a conformational change, and that this interaction does not occur with PI(3,4,5)P_3_. Therefore, as confirmed by the data presented here, the cellular concentration of PI(3,4,5)P_3_ is too low to attract PTEN to the plasma membrane in appreciable amounts.

The H93R and C124S PTEN mutants showed approximately 4-fold stronger binding to PS-bearing membranes compared with *wt* PTEN (Table 2). We have previously suggested that the H93R mutant might bind PS more avidly because this mutation may affect the PS-binding to the C2 domain [38]. Another possibility, however, is that these mutations enhance binding of the phosphatase active site to PS. Since PS has a smaller headgroup than PI(3,4,5)P_3_, it will fit into the active site. However, because it has fewer negative charges and less hydrogen bonding capability, PS would likely have a lower affinity than PI(3,4,5)P_3_. Although speculative at this point, the stronger PS-binding of the mutant proteins in comparison with *wt* PTEN might be due to the fact that PS enters the substrate binding pocket, with the implication that stronger hydrogen bonding (C124S) and stronger ionic interactions as well as hydrogen bonding (H93R) increases the membrane affinity of these mutants.

To assess the role of membrane dynamics in protein binding, we compared the affinities of *wt* PTEN to stBLMs formed from phospholipids with different fatty acid constitutions under otherwise identical conditions (Table 1). For the membrane compositions tested, the results show convincingly that the binding affinity is significantly lower, by a factor of ≈5, for protein binding to DPPC/DPPI(4,5)P_2_ bilayers with low in-plane fluidity than to bilayers with unsaturated chains and, therefore, higher in-plane fluidity. The *B_max_* values for these two situations agree within experimental error, as expected for protein binding in a one-to-one stoichiometry to PI(4,5)P_2_. However, the observed *B_max_* values correspond to protein number densities at the membrane surface that are much lower than the number density of PI(4,5)P_2_. This suggests that either there is not a simple one-to-one stoichiometry in the binding of PTEN to PI(4,5)P_2_ [49] or that local accumulation of PI(4,5)P_2_ in the bilayer, for example driven by electrostatic attraction to adsorbed PTEN molecules, augments the *k_on_* rate of protein binding by increasing the probability for a stable PTEN-PI(4,5)P_2_ interaction in a one-to-one stoichiometry. The latter reasoning follows an argument made by McLaughlin and coworkers to rationalize a similar increase of binding affinity of MARCKS to PI(4,5)P_2_-containing membranes [54] upon membrane fluidization which they attributed to the lateral sequestration through electrostatic interactions of the lipid with protein upon binding. In any case, these results suggest that lateral accumulation of PI(4,5)P_2_, possibly in the form of protein-induced phase-separation, may play a vital role in the binding of the enzyme to the membrane surface. Clearly, more experiments are needed to clarify these details of the binding process, but the binding preference for fluid membranes may very well play an important role for the spatial control of membrane binding *in vivo*, as biomembranes are thought to be mosaics of patches with higher and lower fluidity [55].

Studies of the truncated PTEN variant were primarily undertaken to optimize sample preparation for NR experiments, where the crystal structure [24] will facilitate interpretation of the results. Because of multiple distinctions between *wt* and truncated PTEN, differences in their binding properties are difficult to interpret. In addition, the truncated PTEN has a propensity to aggregate in solution, which precluded binding studies at high protein concentrations. The interaction of the truncated PTEN with PS-containing membranes is more than a factor of two larger than that of the *wt* protein, which could be related to the clipping of the C-terminal tail, 50 AAs in length, which is structurally ill-defined and may tend to obstruct the placement of the *wt* protein at the membrane. Also, the clipped portions of the protein bear a net charge of −14, and therefore their removal in the truncated protein may considerably decrease electrostatic repulsion from the negatively charged membrane surface. Conversely, the association of the truncated PTEN with membranes that contain PI(4,5)P_2_ is somewhat reduced from that observed for the *wt* protein to the same membrane compositions, most likely because of the truncation of the N-terminal segment. Note however, that a key element of the PBM, K13 [35], is present on the truncated PTEN, which lacks only the first 6 AAs [24]. In terms of the amount of bound protein, all of the three membrane compositions studied with the truncated PTEN should be suitable for NR studies, as the *B_max_* values comfortably exceed 60 ng/cm^2^. The main concern for such studies is therefore to have the protein bind in sufficiently high density at the membrane to observe structural differences, while avoiding protein aggregation.

### PTEN Structure on Membranes

**Figure 5:**
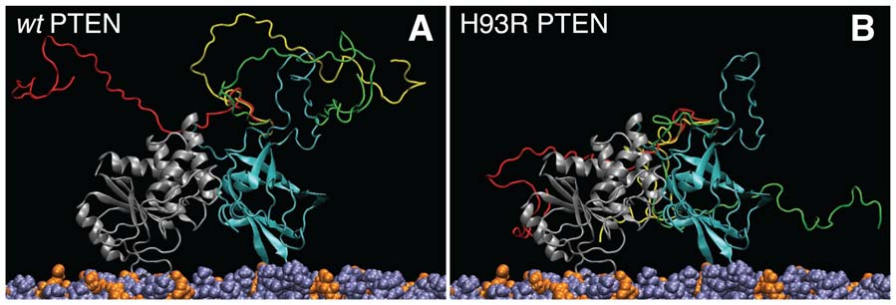
Schematic Depiction of the PTEN Phosphatase on the Surface of a Thermally Disordered stBLM. Peptide backbone representation of (A) *wt* PTEN and (B) H93R PTEN positioned at a DOPC/DOPS (7∶3) membrane surface as deducted from the NR results. The membrane-associated protein penetrates the lipid headgroups (PC: violet, PS: orange) only barely. The PTEN core domains (PD: magenta, C2: grey) are shown in a conformation and membrane orientation deduced from the crystal structure [24]. The close correspondence, observed in Fig. 4, between the nSLD distribution across the interface determined in this work and the nSLD distribution of the truncated PTEN computed from the crystal structure suggests that this is a good approximation. Moreover, about 20% of the protein mass have been deleted in the truncated protein, and ∼20% of the nSLD remains unaccounted for in the overall nSLD distribution for *wt* PTEN in Fig. 4A, if we position the PTEN core domains at the membrane as shown here. The C-terminal tail, which forms the bulk of the deleted peptide, is apparently quite different in its organization in *wt* and H93R PTEN at the membrane. Shown here in red, yellow and green are three distinct conformations, obtained from Monte-Carlo simulations [57], on each PTEN protein core that are consistent with the observed nSLD distributions shown in Figs. 4A and C.

Figure 2 shows that the adsorption of *wt* PTEN to stBLMs leads to significant changes of NR from interface, which can be quantified (Figs. 3 and 4) in terms of contributions of the proteins to the nSLD profiles. It is immediately clear from the protein nSLD distributions that PTEN associates superficially with the membrane and forms monomolecular layers in all cases studied, since the extension of the nSLD increment between the bilayer surface and the bulk buffer corresponds well with the protein size determined in the crystal structure. As quantified in Table 3 and schematically depicted in Fig. 5, any penetration of amino acid sidechains is limited to a few Ångstroms at most. This suggests that the phosphatase goes about its business by scooting peripherally on the membrane surface without penetrating the lipid headgroups. For the interaction of the phosphatase with its substrate, this implies that access of the inositol ring to the PD binding pocket is gained by the lipid pulling out of the plane of the membrane rather than the protein diving into the membrane to engulf the PI(3,4,5)P_3_ headgroup in its binding pocket. In addition, the nSLD distributions of the lipid bilayers before and after PTEN incubation, red and black lines in Fig. 4, respectively, show remarkably little change due to protein association.

**Table 3:** Parameters of the best-fit model for the neutron reflection from stBLMs with associated PTEN proteins.

	*wt* PTEN on PC∶PS = 7∶3 (+chol)	*wt* PTEN on PC∶PS∶PIP_2_ = 68.8∶28.6∶2.6 (+chol)	H93R PTEN on PC∶PS = 7∶3 (+chol)
***Substrate***			
thickness of Au layer / Å	132.5±2.7	157.7±0.3	129.5±1.4
nSLD of Au layer / 10^−6^ Å^−2^	4.44±0.01	4.47±0.02	4.50±0.01
substrate roughness / Å	4.5±0.5	4.3±0.4	3.9±0.6
***Sparsely-Tethered Bilayer Lipid Membrane***			
area per phospholipid (as-prepared stBLM) / Å^2^	66.2±1.8	59.8±1.0	61.1±0.9
thickness of tether layer / Å	18.8±0.4	16.6±0.4	19.1±0.4
thickness of lipid bilayer core / Å	28.9±0.4	29.6±0.5	30.1±0.3
thickness of lipid headgroup / Å	9.6 (fixed)	9.6 (fixed)	9.6 (fixed)
thermal bilayer roughness / Å	3.4±0.4	5.0±0.3	3.0±0.1
***PTEN Protein***			
PTEN penetration into lipid bilayer / Å	4.3±2.7	0.1±2.0	2.4±3.3
total amount of adsorbed protein in H_2_O-based buffer (volume per surface area) / Å	6.8±1.0	7.3±0.9	3.0±0.8
fraction of isotopically exchanged protons	0.69±0.13	0.68±0.14	0.73±0.18
distance of center of mass of protein from bilayer interface / Å	23.9±1.9	29.6±1.4	25.1±5.2
***Global Properties***			
quality of best-fit (χ^2^)	1.68	2.86	2.22

Error limits indicate 68% confidence intervals derived from Monte-Carlo data resampling of the data [39].

Both experiments with *wt* PTEN–adsorption to an stBLM that contains only PC and PS and to an stBLM that also contains 2.6 mol% PI(4,5)P_2_–show almost identical nSLD distributions [56]. If there is any distinction, then the protein hugs the membrane more closely on the PS-only bilayer and also, the nSLD distributions at *d*>100 Å appear slightly different. In both cases, the correspondence of the free-form fit of the experimental data with the nSLD distribution derived from the crystal structure of the truncated PTEN is striking. The major portion of the protein nSLD near the bilayer surface is almost perfectly described by the crystal structure, while there are ∼15–20% of the nSLD, distally located from the membrane surface, unaccounted for in the experimental nSLD distributions. We draw the following conclusions for the structural interpretation of these results: (1) Because the putative nSLD distribution in the crystal structure was determined by assuming the orientation of the protein on the membrane as proposed by Lee and coworkers [24], and different orientations would result in significantly different nSLD distributions, the close correspondence suggests strongly that the C2 and phosphatase domains are organized as determined in the crystal structure and are both in close association with the bilayer of the stBLM. The resulting membrane positioning consistent with the nSLD profiles is schematically shown for *wt* and H93R PTEN in Figs. 5A and B, respectively. (2) FTIR characterization of dissolved and membrane-bound *wt* PTEN showed changes in the secondary structure upon membrane binding [35]. Our NR data suggest that these structural changes are indeed minor if they affect the C2 and phosphatase domains or that they occur in those parts of the protein that were clipped in the crystal structure, for example, on the ∼50 AA long C-terminal tail. (3) Given the close correspondence between the protein region proximal to the bilayer and the crystal structure, it is tempting to assume that (the major portion of) the nSLD which is unaccounted for in the distal region is due to the truncated stretches on the crystal structure. While the truncation on the N-terminal tail is short and the clipped stretch within the C2 domain is fixed in its location on the protein, the long stretch of C-terminal tail is not similarly confined and could be rather mobile. We propose that the extra nSLD in the experimental ρ*_n_* profile is associated with this C-terminal stretch which points away from the membrane-bound protein with an overall conformation that is still in close vicinity to the two major protein domains, C2 and PD (Fig. 5). While the sheer length of the C-terminal tail would permit it to reach the membrane surface and interact with the lipids, the observed nSLD profile suggests that this is not the case. However, it will require further efforts to establish the conformation of the C-terminal tail more rigorously, for example with NR experiments using *wt* PTEN protein with the C-terminus specifically deuterated.

The analysis of the reflectivity results is somewhat preliminary because we haven't yet had the opportunity to conduct NR investigations with varying protein concentrations or studies using specific deuteration. In particular, experiments with deuterated PTEN will unmistakably show the spatial organization of, for example, the C-terminal protein stretch. The current data refinement yields *envelopes* of nSLD for PTEN, without any direct reference to the protein's internal structure. Because the nSLD distribution of the truncated PTEN is not pronouncedly asymmetric about its peak density, there is the possibility that the bound protein is not unidirectionally oriented at the membrane. A distribution of up-down orientations is, on the other hand, an unlikely scenario because the protein-membrane interaction is expected to be grossly different for the membrane-proximal and membrane-distal faces of the protein. Therefore, the two orientations would likely have different distances from the membrane surface, and one would expect a significantly broadened distribution from that predicted by the crystal structure, which we do not observe.

As functionality assays and our SPR results show, the point mutation in H93R PTEN affects its catalytic affinity and membrane binding to PS- and (PS+PIP(4,5)P_2_)-containing membranes to a surprisingly high extent, given that it differs from the *wt* protein only by one single AA. It may therefore be less surprising that the H93R mutant also shows a significantly different nSLD distribution at the membrane surface. There are several reasons that could lead to these differences. In line with our interpretation of the *wt* PTEN nSLD distributions discussed above, the lack of nSLD in the distal region of the H93R protein may reflect a distinct organization of the C-terminal tail. As Fig. 4c shows, the *extension* of H93R PTEN at the membrane matches the overall width of the nSLD distribution derived for the truncated PTEN crystal structure quite well–better, in fact, than the nSLD distributions of *wt* PTEN. On the other hand, the *detailed profile* of the truncated PTEN is not well matched by the H93R PTEN nSLD distribution. This would be expected if the C-terminal tail, which provides the bulk of nSLD unaccounted for in the crystal structure, is located at the same distance from the membrane surface as the major domains of the membrane-bound PTEN, *e.g.*, at *d*∼90 Å, where as it is further away from the membrane for the *wt* protein, at *d*>100 Å. In any case, without further information about the conformation of this part of the protein, for example from NR investigations of specifically deuterated PTEN, such structural details cannot be resolved with confidence.

While the differences between the structures of *wt* and H93R PTEN on the membrane deserve further investigation, we summarize our NR results as showing conclusively that the PTEN phosphatase scoots along the membrane in search of its PI(3,4,5)P_3_ substrate molecules and most likely pulls their headgroups slightly out of the membrane surface to gain access with its catalytic binding pocket. Electrostatic, and possibly also chemically selective interactions of the C2 domain with PS help pull the phosphatase tight to the membrane surface to augment its interaction with the substrate. The PTEN protein goes about its business without penetrating the lipid headgroups in the bilayer. In doing so, the two major domains of the protein sit flush on the membrane surface and the regulatory C-terminal tail is most likely pushed out of the way, located on the distal side of the protein core.

This work provides a wealth of new information about the membrane association and likely mode of action of the PTEN phosphoinositide phosphatase. We show that binding of the enzyme to the membrane surface is synergistically affected by two membrane components, PS and PI(4,5)P_2_. Membrane fluidity is an important factor in determining the protein's affinity to the membrane, which we suggest is due to the binding of multiple PI(4,5)P_2_ lipids to the protein which actively recruits them through attractive interactions. The mutations C124S and H93R both affect the PTEN membrane affinity significantly. For C124S that can be rationalized by the fact that this mutant is incapacitated in its catalytic activity. The reasons for the rather dramatic effects documented for H93R, which binds with much higher affinity to PS but shows a reduction of its affinity to PI(4,5)P_2_, are less clear.

NR studies of the PTEN phosphatase on the in-plane fluid, thermally disordered bilayer membrane provide a first glimpse into the structural details of an active phosphoinositide phosphatase at the membrane surface and start to fill in an emerging picture of the structure-function relationship. We observe that PTEN scoots along the membrane superficially without penetration the lipid headgroup. Because of the lack of membrane penetration, the phosphatase probably does not even reach the position of the lipid headgroup phosphates while surfing the membrane. In view of the depth of its catalytic binding pocket, it is likely that the substrate is somewhat pulled out of the membrane, presumably through electrostatic interactions, to promote access to the inositol ring. Our current interpretation of the overall nSLD distribution of the protein at the interface is that the regulatory C-terminal tail is pushed away from the bilayer to the distal region of the membrane-bound protein.

## Supporting Information

Information S1 **The Information S1 provides a schematic structure of the tether lipid, HC18; the procedure used to calibrate the custom-made SPR instrument; the procedure used to estimate** ***K_d_*** **ranges from insufficient SPR data sets; and the full sets of neutron reflectivity data measured for the various systems that have been investigated, as well as the derived nSLD profiles.** A full set of model parameters used to describe the neutron reflectivity data sets is also given.
(PDF)
