# Supplementary material for: Membrane Association of the PTEN Tumor Suppressor: Molecular Details of the Protein-Membrane Complex from SPR Binding Studies and Neutron Reflection
Source: PLoS One. 2012 Apr 10;7(4):e32591. doi: 10.1371/journal.pone.0032591 (PMC3323581; doi:10.1371/journal.pone.0032591)
Supplement: Information S1 — The Information S1 provides a schematic structure of the tether lipid, HC18; the procedure used to calibrate the custom-made SPR instrument; the procedure used to estimate Kd ranges from insufficient SPR data sets; and the full sets of neutron reflectivity data measured for the various systems that have been investigated, as well as the derived nSLD profiles. A full set of model parameters used to describe the neutron reflectivity data sets is also given. (PDF) [file pone.0032591.s001.pdf]

Supporting Information: Shenoy *et al.*,
Membrane Association of the PTEN Tumor Suppressor

I. Molecular structure of HC18

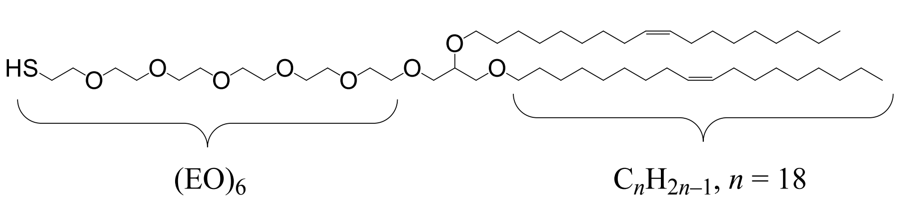

Z 20-(*Z* octadec-9-enyloxy)-3,6,9,12,15,18,22-heptaoxatetracont-31-ene-1-thiol (HC18)

Figure S1: **Structure of HC18.**

II. Calibration of SPR Instrument

1 Conversion between Pixels and Degrees

glycerol in water (% by volume)	refractive index (*n*)
0	1.32972
1	1.33145
2	1.33317
3	1.33488
4	1.33658
5	1.33828
6	1.33996
7	1.34164
8	1.34330
9	1.34496
10	1.34661

Table S1: **Index of Refraction of Glycerol Solutions.** Mass-weighted refractive index of glycerol-water mixtures.

Standard SPR reflectivity curves are plots of the reflectivity as a function of incidence angle in degrees. As our SPR uses a fan-design, it samples a range of incidence angles simultaneously.[1] However, the angles are reported as pixels on the detector and a conversion fac-

tor from pixels to degrees needs to be calculated for comparison of our data to the literature. This was accomplished by using glycerol:water mixtures of known refractive index on slides of known gold and chromium thickness thereby allowing for a comparison between the SPR minimum in pixels (experimental) and the SPR minimum in degrees (theoretical).[2]

While the refractive index of glycerol:water mixtures are commonly tabulated in the literature and in handbooks, we were unable to find any corresponding to λ = 763.8 nm, the wavelength used in our SPR. Weighting the refractive index of water and glycerol by the respective volume fractions has been shown to be a bad estimate of the refractive index of the mixture.[3] Instead, we resorted to weighting the water and glycerol components by their respective mass fractions. At λ = 763.8 nm, the refractive index of pure glycerol is 1.46716 (Ref. 4) and that of water is 1.32972.[5] Glycerol:water mixtures were prepared ranging from 0% glycerol (by volume) to 10% glycerol in 1% increments. The mass-weighted refractive indices of these mixtures are given in Table S1.

Using the Fresnel model, we can fit the SPR reflectivity curve to obtain the refractive index and thickness of each layer of dielectric medium on the glass prism. For the substrate used in this calibration measurement, the parameters are given in Table S2.

substrate	*n*	*k*	thickness (nm)
glass slide	1.52	–	–
chromium	3.095	3.428	5
gold	0.172	4.7361	44

Table S2: **Substrate Parameters.** *n* and *k*, respectively, are the real and imaginary parts of the complex refractive index.

glycerol in water (% by volume)	SPR minimum (deg)
0	65.9751
1	66.1537
2	66.3327
3	66.5117
4	66.6910
5	66.8716
6	67.0512
7	67.2322
8	67.4123
9	67.5937
10	67.7753

Table S3: **Calibration Experiment.** Expected SPR minimum for a given glycerol:water mixture on the given substrate.

From the data given in Tables S1 and S3, we obtain the following conversion relation:

$$\Delta n = 105.8 \pm 0.2 \text{ deg} \quad \text{(Eq. S1)}$$

This gives a relation connecting the change in refractive index and the corresponding SPR minimum in degrees. In order to calculate the conversion factor between refractive index change and the SPR minimum in pixels, we performed 3 calibration experiments detailed in Tables S4, S5 and S6.

glycerol in water (% by volume)	SPR minimum (in pixels)
0	292.640 ± 0.002
2	348.703 ± 0.004
4	410.500 ± 0.004
6	462.840 ± 0.004
8	510.260 ± 0.003
10	542.790 ± 0.004

Table S4: **Calibration Experiment 1**. Experimental SPR minimum (in pixels) for a given glycerol:water mixture on the given substrate.

glycerol in water (% by volume)	SPR minimum (in pixels)
0	287.650 ± 0.004
1	308.880 ± 0.001
2	334.280 ± 0.001
3	361.160 ± 0.005
4	389.060 ± 0.002
5	423.910 ± 0.004

Table S5: **Calibration Experiment 2**. Experimental SPR minimum (in pixels) for a given glycerol:water mixture on the given substrate.

glycerol in water (% by volume)	SPR minimum (in pixels)
1	273.030 ± 0.004
2	296.700 ± 0.004
3	327.730 ± 0.004
4	368.760 ± 0.004
5	396.740 ± 0.004

Table S6: **Calibration Experiment 3**. Experimental SPR minimum (in pixels) for a given glycerol:water mixture on the given substrate.

From Table S4 we obtain:

$$\Delta\ 1\ \text{pixel} = (6.11 \pm 0.08) \times 10^{-5}\ \Delta n \qquad \text{(Eq. S2)}$$

From Table S5 we obtain:

$$\Delta\ 1\ \text{pixel} = (6.47 \pm 0.06) \times 10^{-5}\ \Delta n \qquad \text{(Eq. S3)}$$

From Table S6 we obtain:

$$\Delta\ 1\ \text{pixel} = (6.4 \pm 0.3) \times 10^{-5}\ \Delta n \qquad \text{(Eq. S4)}$$

Averaging Eqns. S2, S3 and S4, we obtain the relation:

$$\Delta\ 1\ \text{pixel} = (6.3 \pm 0.1) \times 10^{-5}\ \Delta n \qquad \text{(Eq. S5)}$$

From Eqns. S1 and S5, we obtain the relation:

$$\Delta\ 1\ \text{pixel} = 0.0067 \pm 0.0001\ \text{deg} \qquad \text{(Eq. S6)}$$

Given an SPR minimum in pixels, we calculate the absolute SPR minimum in degrees as:

$$\text{SPR Minimum (deg)} = (63.97 \pm 0.03) + (0.0067 \pm 0.0001) \times \text{SPR minimum (pixels)} \qquad \text{(Eq. S7)}$$

2 Relation between SPR Minimum Change and Protein Layer Thickness and Surface Density

The Fresnel model can be used to fit the refractive index and thickness of a lipid bilayer atop the thin gold film on the glass slide. However, fitting a protein layer atop a bilayer atop a substrate has sufficiently high number of parameters such that an accurate fit of the parameters is nearly impossible.

Instead, it is easier to calculate a conversion factor between SPR minimum change and protein thickness (or surface density) as observed by comparing the reflectivity curve corresponding to the bilayer with that for the bilayer with protein.

The relation between protein thickness and surface density is given by:[6]

$$\Gamma = \frac{d_{protein}\left(n_{protein} - n_{bulk}\right)}{\mathrm{d}n/\mathrm{d}c} \qquad \text{(Eq. S8)}$$

where Γ is the surface density, $d_{protein}$ is the thickness of the protein layer, $n_{protein}$ is the refractive index of the protein, n_{bulk} is the refractive index of the bulk buffer and dn/dc is the change in refractive index of the protein as a function of protein concentration.

The standard value for $n_{protein}$ is 1.41, for n_{lipid} is 1.5 and for n_{bulk} is 1.33. $dn/dc = 0.187 \pm 0.003$ ml/g was calculated for Bovine Serum Albumin (BSA).[6] Plugging these values into Eq. S8, we obtain:

$$\Gamma = 4.3 \times d_{protein}\ \mathrm{ng/cm^2} \qquad \text{(Eq. S9)}$$

where $d_{protein}$ is in Å.

We can assume the same substrate parameters as in Table S2. There are at least three distinct ways to model a tethered bilayer lipid membrane (tBLM) in a slab model:

1. Single slab model: We can consider the “tether layer” (which actually contains the EO_n tether and βME), plus the lipid chains and headgroups as one single slab with a thickness of d = 55 Å and index, n = 1.5.
2. Double slab model: Treat the “tether layer” and the lipid chains as a slab of d = 45 Å and n = 1.5, and the distal headgroups as a slab of d = 10 Å and n = 1.417 (assuming 50% lipid and 50% water by volume).
3. Triple slab model: Treat the “tether layer” as a slab of d = 15 Å and n = 1.442 (65% PEG and 35% water), the lipid chains as a slab of d = 30 Å and n = 1.5 and the distal headgroups as a slab of d = 10 Å and n = 1.417 (assuming 50% lipid and 50% water by volume).

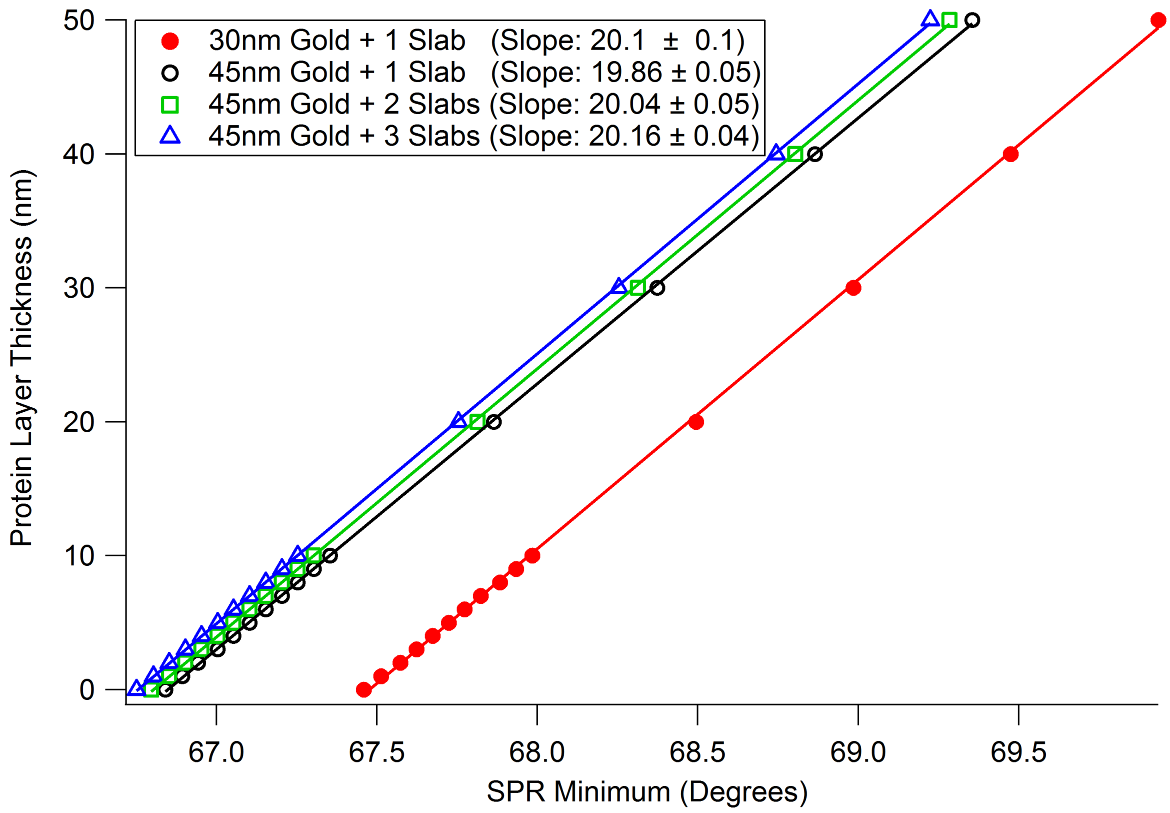

<u>Figure S2:</u> **SPR Calibration.** Correlation of protein film thickness with SPR minimum position in different model descriptions of the interfacial structure.

For each model, we plot a theoretical curve of the neat bilayer and then add increasing amounts of protein (n = 1.41). We then plot the SPR minimum as a function of protein layer

thickness. We did this for all three models and also for model 1 (assuming 30 nm of gold instead of the standard 45 nm) to test the effect of gold thickness (see Fig. S1).

In all four cases, the slope obtained was approximately 20 nm/deg. This means that a change in the SPR minimum of the reflectivity profile between the bilayer and (bilayer + protein) corresponds to the addition of a 20 nm protein layer. Given the conversion relation between pixels and degrees (Eq. S7), we obtain:

$$\Delta 1 \text{ pixel} \Rightarrow \Delta d_{protein} = 0.134 \text{ nm} \qquad \text{(Eq. S10)}$$

i.e., a 1 pixel increase in the SPR minimum corresponds to the addition of 0.134 nm of (homogeneously distributed) protein.

$$\Delta d_{protein} = 1 \text{ nm} \Rightarrow \Delta(\text{SPR minimum}) = 7.5 \text{ pixels} \qquad \text{(Eq. S11)}$$

i.e., the addition of a 1 nm layer of protein corresponds to an increase in the SPR minimum by 7.5 pixels.

Combining Eqns. S10 and S11, finally yields the relation:

$$\Delta 1 \text{ pixel} \Rightarrow \Delta\Gamma = 5.8 \text{ ng/cm}^2 \qquad \text{(Eq. S12)}$$

i.e., a 1 pixel increase in the SPR minimum corresponds to an increase in protein surface density by 5.8 ng/cm^2.

III. Estimate of the K_d from Insufficient SPR Data. Example for the Binding of the Truncated PTEN Mutant to PS-Containing Membranes

The equilibrium binding constant (K_d) can be determined with high precision if the highest concentration of protein used in the SPR experiment is sufficiently greater than the K_d. For binding of the truncated PTEN to PS containing membranes, we were limited to a maximum protein concentration of 3 μM due to aggregation. However, this concentration appeared to be sufficiently close to the Kd that we were able to develop a criterion to estimate its value and define confidence limits.

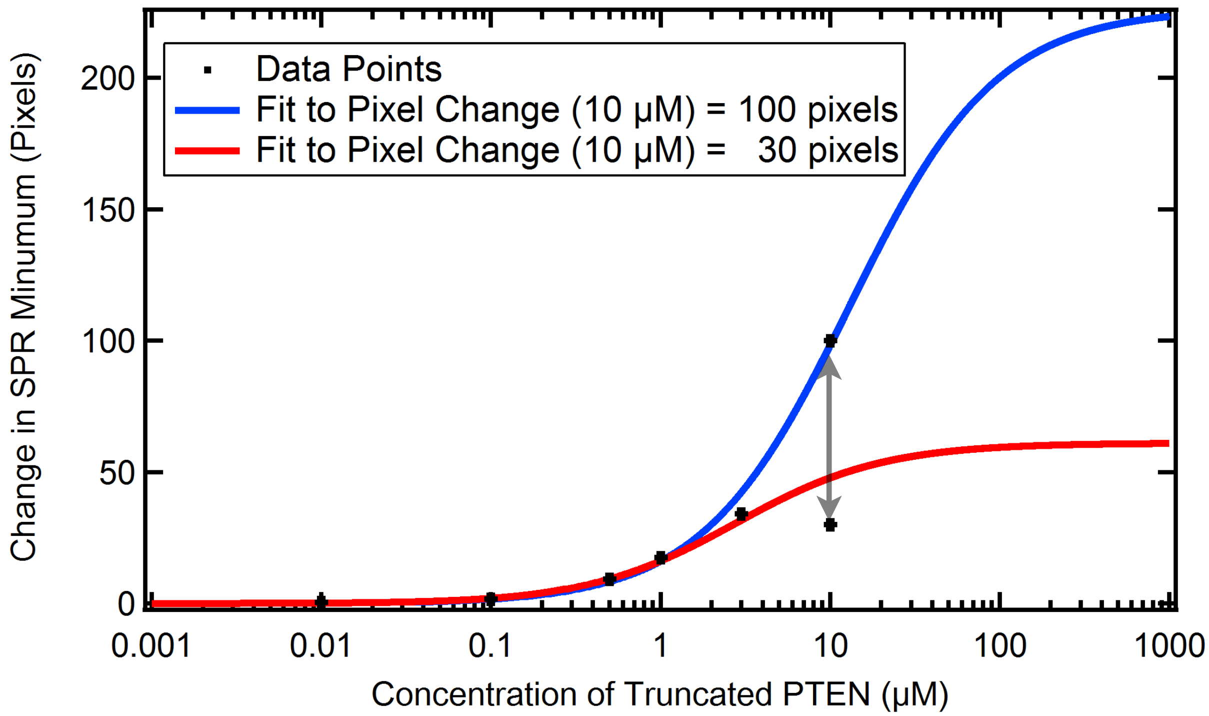

Figure S3: **Fits to an SPR Data Set with Insufficient Data.** SPR data sets for with high protein concentrations are not available were evaluated by estimating the *possible* range of K_d and B_{max} values. In this example, the SPR minimum change for a fictitious data point at a protein concentration of 10 μM was varied between 30 and 100 pixels (grey arrow). Two fits assuming a Langmuir adsorption isotherm shown in blue and red represent these extremal values of the fictitious data point.

The 3 μM concentration corresponds to an experimentally measured change in the SPR reflectivity minimum of 34.0 ± 0.2 pixels (as compared to the baseline bilayer signal). We added a fictitious data point at 10 μM and varied its position on the ordinate from 30 pixels to 100 pixels, both significantly larger than the 0.2 pixel standard deviation in 1 pixel increments. We then fitted each data set (that now consisted of 5 experimentally determined points and 1 fictitious data point) to determine the K_d and the quality of the fit, χ^2 (see Fig. S4).

To account for the uncertainties in protein concentration and pixel change associated with the experimental data points, a Monte-Carlo resampling method was used.[7] From the error bars for each data point, 1000 statistically independent data sets were generated any of which could have occurred given the statistical uncertainties. For each of the 1000 data sets, we varied the pixel change for the 10 µM concentration from 30 pixels to 100 pixels and calculated the K_d and χ^2. Figure S4 shows the averaged K_d and χ^2 values for a given pixel change at 10 µM protein concentration across all 1000 fits and their uncertainties (68% confidence limits). The smallest χ^2 of 10.7 corresponds to a K_d of 3.3 µM. Again using one standard deviation, we determine bounds on K_d of 2.5 µM to 4.9 µM.

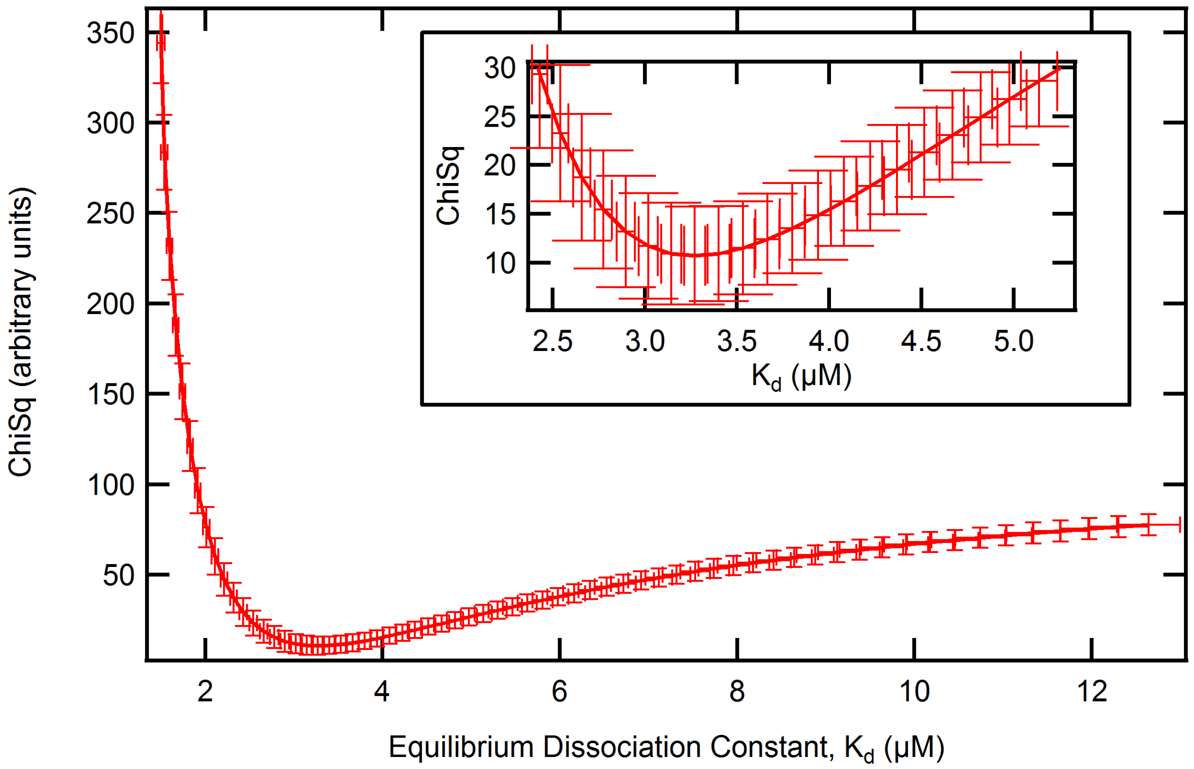

<u>**Figure S4:**</u> **Estimate of a Range of K_d Values from Insufficient Data.** A plot of χ^2 and K_d, averaged over 1000 Monte-Carlo resampled data sets, as the pixel change for the 10 µM concentration is varied from 30 to 100 pixels. Inset: Magnified view of the χ^2 region around the minimum.

IV. Neutron Reflectivities and nSLD Profiles: Full Data Sets

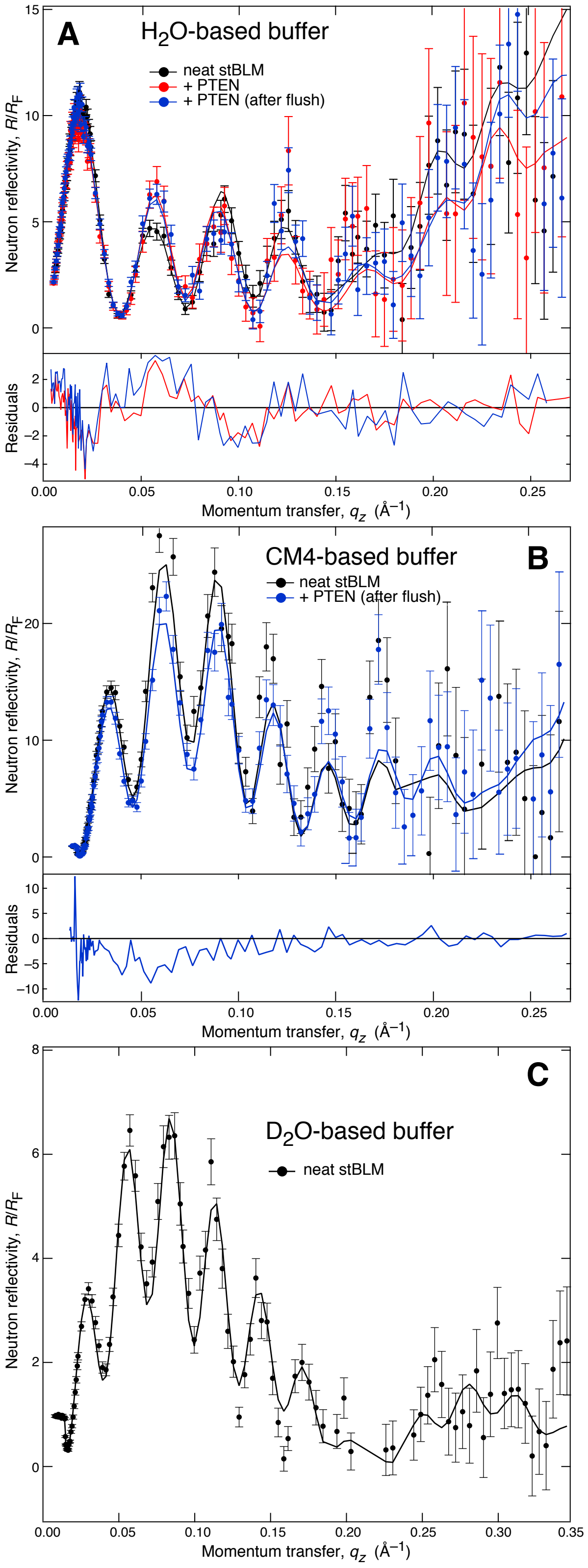

<u>Figure S5:</u> Neutron Reflectivity, Normalized to the Fresnel Reflectivity, of *wt* PTEN Bound to an stBLM with ~ 30 mol% PS. (A) NR spectra and best fits of a DOPC: DOPS:chol = 70:30:5 stBLM in H_2O-based buffer **1** prepared on a Si substrate with a Cr bonding layer and a terminal Au film. Black: as-prepared stBLM; red: stBLM in buffer **1** with 20 μM *wt* PTEN; blue: stBLM in buffer **1** without protein (loosely adsorbed protein flushed off). Changes of the spectra against that of the as-prepared stBLM are shown as residuals, normalized to the magnitude of the experimental errors, at the bottom. (B) NR spectra and best fits for the same stBLM in CM4-based buffer **1**. Black: as-prepared stBLM (continuation of the experiment shown in black in panel A); blue: stBLM in buffer **1** without protein after the adsorption of protein in H_2O-based buffer **1** (continuation of the experiment shown in blue in panel A). Changes of the spectrum against that of the as-prepared stBLM are shown as residuals, normalized to the magnitude of the experimental errors, at the bottom. (C) NR spectrum and best fit for the same (as-prepared) stBLM in D_2O (continuation of the experiment shown in black in panel B). No protein data set was measured at this contrast.

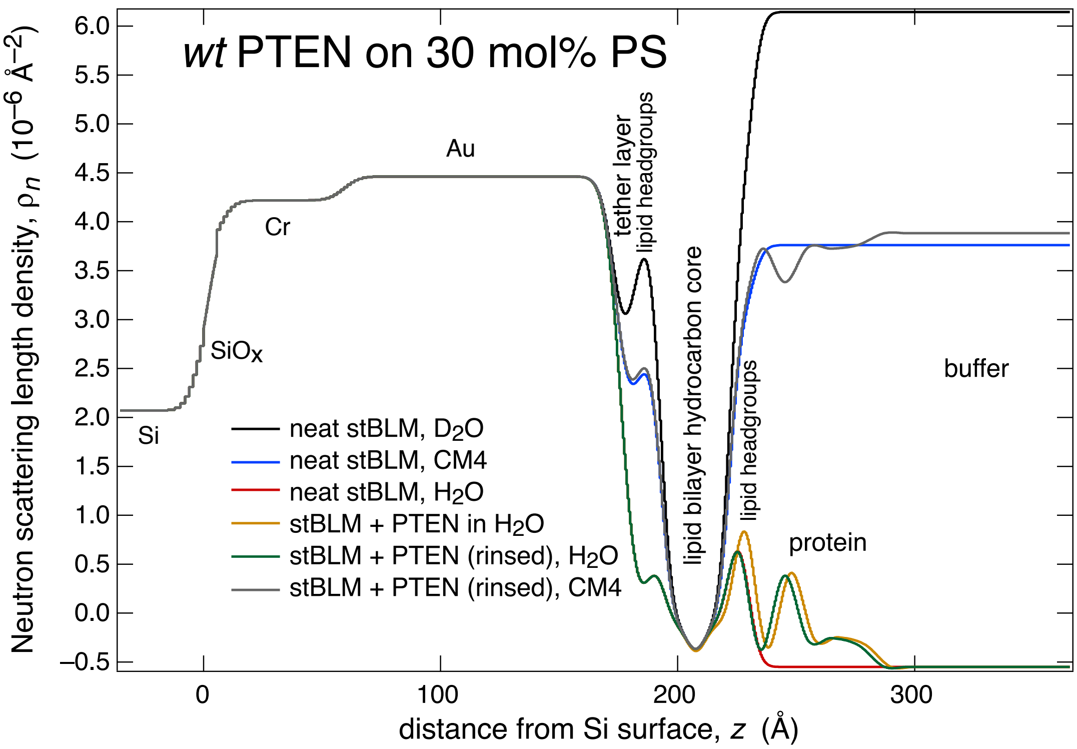

Figure S6: **Scattering Length Density Distributions Derived from NR Data.** nSLD profiles derived from the joint refinement (Continuous Distribution model) of the data shown in Fig. S5 for *wt* PTEN bound to an stBLM with ~ 30 mol% PS. The substrate consists of a Si wafer with its natural oxide (SiO_x) layer that carries a Cr bonding layer and a terminal Au film.

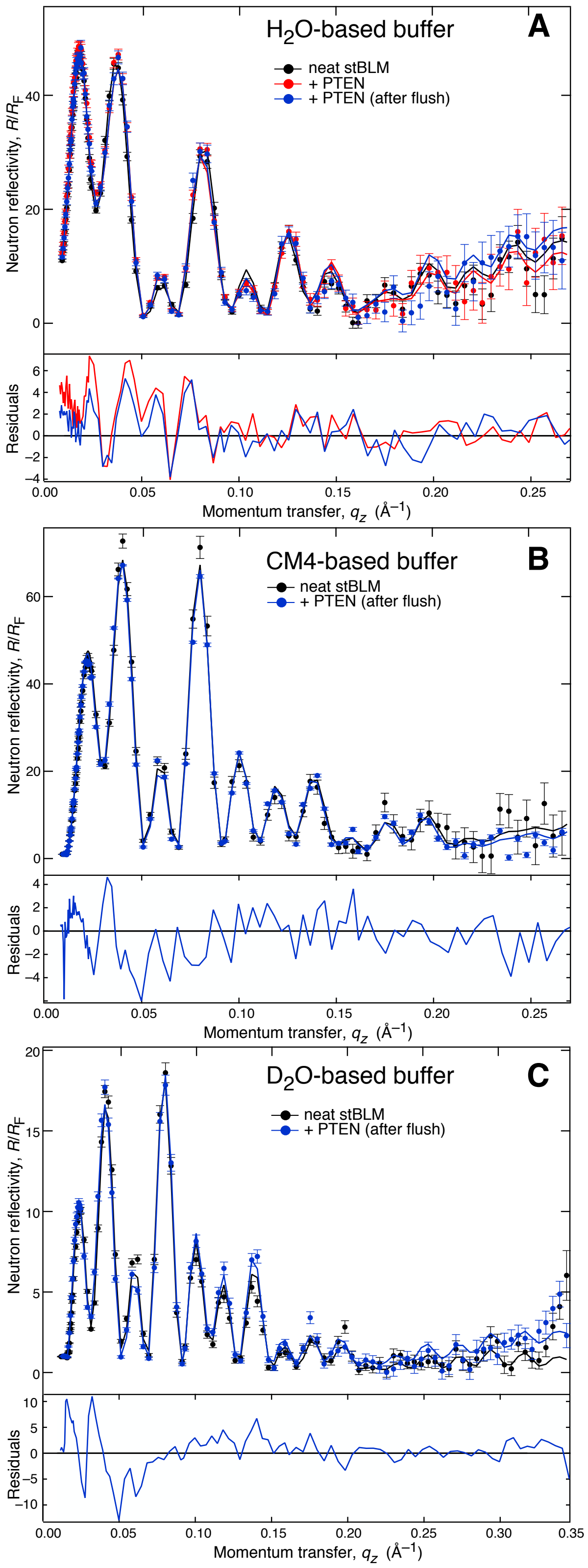

<u>Figure S6:</u> Neutron Reflectivity, Normalized to the Fresnel Reflectivity, of *wt* PTEN Bound to an stBLM with (PS + PI(4,5)P$_2$). (A) NR spectra and best fits of a DOPC: DOPS:PI(4,5)P$_2$:chol = 70:30:5 stBLM in H_2O-based buffer **1** prepared on a Si substrate with a Fe-Ni bonding layer and a terminal Au film. Black: as-prepared stBLM; red: stBLM bathed in buffer **1** with 20 µM *wt* PTEN; blue: stBLM in buffer **1** without protein (loosely adsorbed protein flushed off). Changes of the spectra against that of the as-prepared stBLM are shown as residuals, normalized to the magnitude of the experimental errors, at the bottom. (B) NR spectra and best fits for the same stBLM in CM4-based buffer **1**. Black: as-prepared stBLM (continuation of the experiment shown in black in panel A); blue: stBLM in buffer **1** without protein after the adsorption of protein in H_2O-based buffer **1** (continuation of the experiment shown in blue in panel A). Changes of the spectrum against that of the as-prepared stBLM are shown as residuals, normalized to the magnitude of the experimental errors, at the bottom. (C) NR spectra and best fits for the same stBLM in D_2O-based buffer **1**. Black: as-prepared stBLM (continuation of the experiment shown in black in panel B); blue: stBLM in buffer **1** without protein after the adsorption of protein in H_2O-based buffer **1** (continuation of the experiment shown in blue in panel B). Changes of the spectrum against that of the as-prepared stBLM are shown as residuals, normalized to the magnitude of the experimental errors, at the bottom.

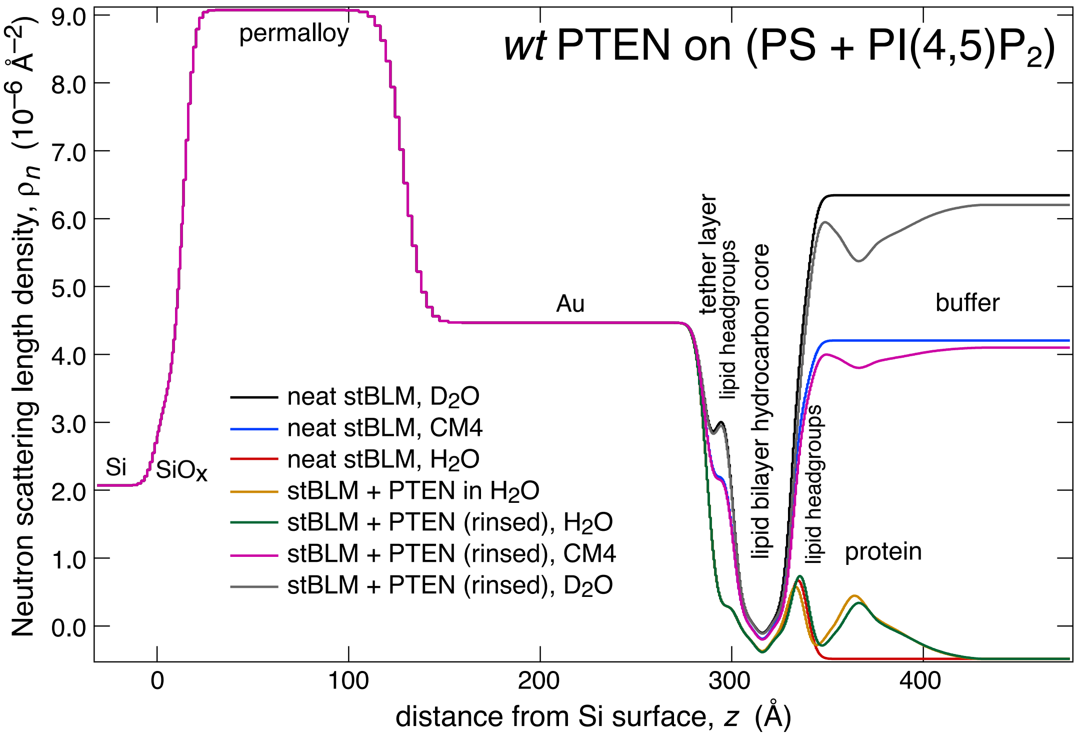

<u>Figure S8:</u> **Scattering Length Density Distributions Derived from NR Data.** nSLD profiles derived from the joint refinement (Continuous Distribution model) of the data shown in Fig. S7 for *wt* PTEN bound to an stBLM with (PS + PI(4,5)P_2). The substrate consists of a Si wafer with its natural oxide (SiO_x) layer that carries a Fe-Ni (permalloy) bonding layer and a terminal Au film.

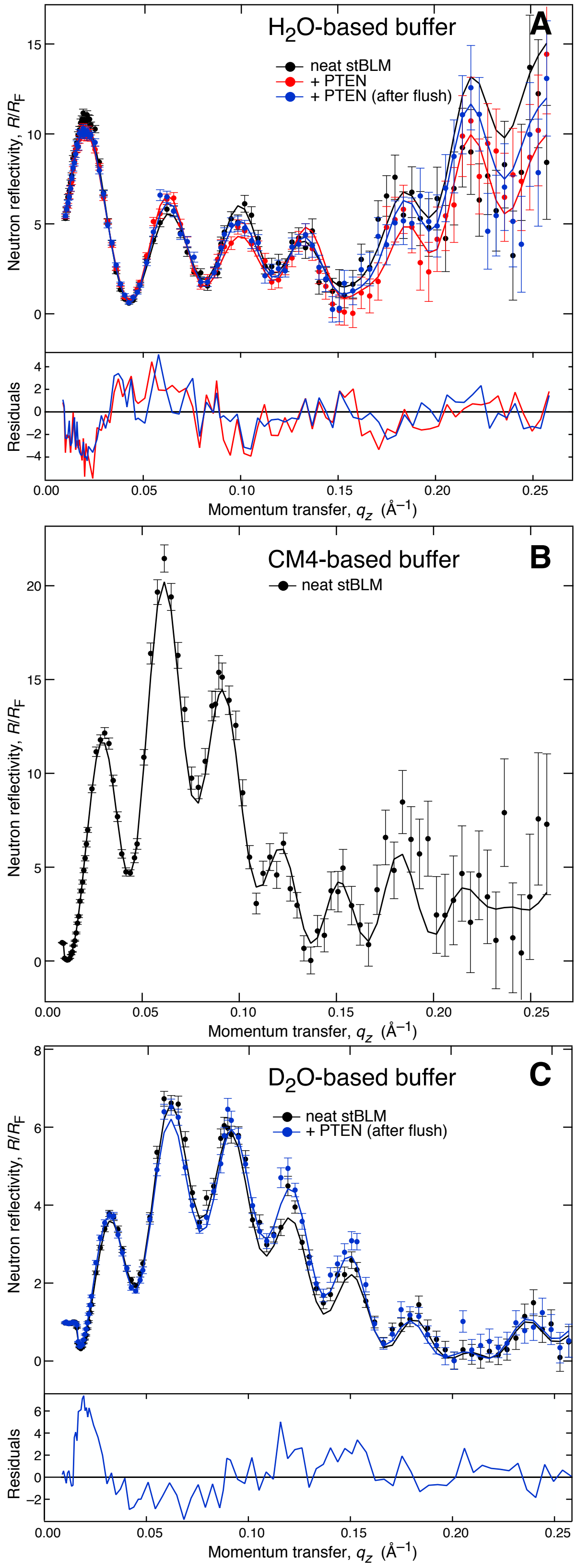

<u>Figure S9:</u> Neutron Reflectivity, Normalized to the Fresnel Reflectivity, of H93R PTEN Bound to an stBLM with ~ 30 mol% PS. (A) NR spectra and best fits of a DOPC:DOPS:chol = 70:30:5 stBLM in H_2O-based buffer **1** prepared on a Si substrate with a Cr bonding layer and a terminal Au film. Black: as-prepared stBLM; red: stBLM in buffer **1** with 15 μM H93R PTEN; blue: stBLM in buffer **1** without protein (loosely adsorbed protein flushed off). Changes of the spectra against that of the as-prepared stBLM are shown as residuals, normalized to the magnitude of the experimental errors, at the bottom. (B) NR spectrum and best fit for the same stBLM in CM4-based buffer **1** (continuation of the experiment shown in black in panel A). No protein data set was measured at this contrast. (C) NR spectra and best fits for the same stBLM in D_2O-based buffer **1**. Black: as-prepared stBLM (continuation of the experiment shown in black in panel B); blue: stBLM in buffer **1** without protein after the adsorption of protein in H_2O-based buffer **1** (continuation of the experiment shown in blue in panel A). Changes of the spectrum against that of the as-prepared stBLM are shown as residuals, normalized to the magnitude of the experimental errors, at the bottom.

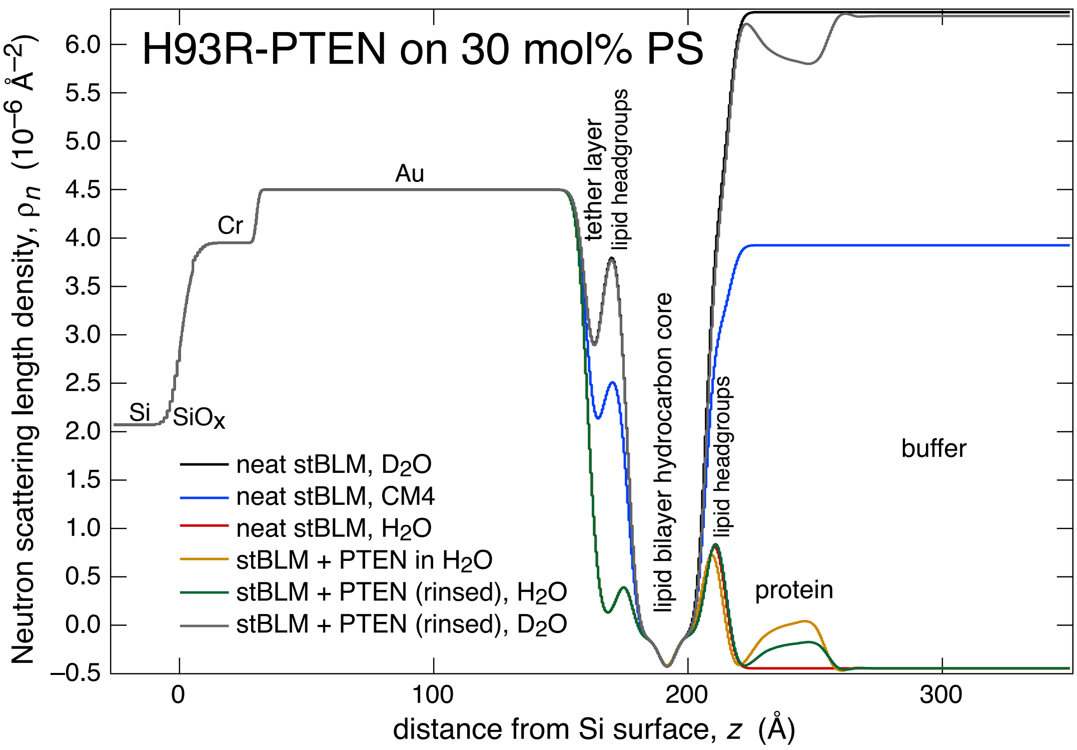

Figure S10: **Scattering Length Density Distributions Derived from NR Data.** nSLD profiles derived from the joint refinement (Continuous Distribution model) of the data shown in Fig. S9 for H93R PTEN bound to an stBLM with ~ 30 mol% PS. The substrate consists of a Si wafer with its natural oxide (SiO_x) layer that carries a Cr bonding layer and a terminal Au film.

V. Full Set of Fit Parameters for the Neutron Reflection Data Sets

	wt PTEN PC:PS (+ chol)	*wt* PTEN PC:PS:PIP(4,5)P_2 (+ chol)	H93R PTEN PC:PS (+ chol)
**Substrate**			
thickness of SiO_x / Å	5.0 ± 0.5	13.3 ± 0.6	5.4 ± 0.9
nSLD of SiO_x / 10^{-6} Å^{-2}	3.55 (fixed)	3.47 (fixed)	3.55 (fixed)
thickness of bonding layer / Å	chromium 38.0 ± 2.5	permalloy 114.9 ± 0.2	chromium 24.92 ± 1.3
nSLD of bonding layer* / 10^{-6} Å^{-2}	4.12 ± 0.03	9.08 ± 0.01	3.95 (fixed)
roughness of bonding layer / Å	4.5 ± 0.6	8.3 ± 0.2	3.4 ± 2.5
thickness of Au layer / Å	133.0 ± 2.6	157.6 ± 0.2	130.0 ± 0.9
nSLD of Au layer / 10^{-6} Å^{-2}	4.44 ± 0.01	4.45 ± 0.02	4.50 ± 0.01
substrate roughness / Å	4.5 ± 0.6	4.8 ± 0.3	3.7 ± 0.6
**Tethered Lipid Bilayer**			
thickness of tether layer / Å	18.8 ± 0.4	16.0 ± 0.3	18.9 ± 0.3
thickness of hydrocarbon core of lipid bilayer / Å	28.8 ± 0.4	30.2 ± 0.4	30.1 ± 0.3
completeness of bilayer	1.00 ± 0.01	0.96 ± 0.01	1.00 ± 0.00
bilayer roughness / Å	3.4 ± 0.4	3.8 ± 0.5	3.0 ± 0.1
thickness change of hydrocarbon core while protein is incubating / Å	1.2 ± 1.5	-0.6 ± 1.0	0.1 ± 1.1
thickness change of hydrocarbon core after buffer rinse / Å	0.0 ± 0.4	1.0 ± 0.2	0.2 ± 0.3
molar fraction of tether molecules in inner lipid leaflet	0.64 ± 0.11	0.99 ± 0.11	0.86 ± 0.14
number of βME backfiller molecules per tether molecule	3.5 ± 0.9	1.5 ± 0.3	1.5 ± 0.7
area per lipid (neat bilayer)** / Å^2	65.9 ± 0.8	62.9 ± 0.7	62.0 ± 0.5

	wt PTEN PC:PS (+ chol)	*wt* PTEN PC:PS:PIP(4,5)P_2 (+ chol)	H93R PTEN PC:PS (+ chol)
**Protein**			
penetration into lipid bilayer / Å	4.3 ± 2.6	0.5 ± 2.2	2.4 ± 3.3
fraction of accessible exchangeable protons	0.59 ± 0.23	0.60 fixed	0.60 fixed
fraction of protein remaining at the membrane after first rinse	0.65 ± 0.11	0.99 ± 0.10	0.70 ± 0.17
fraction of protein remaining at the membrane after second rinse	0.55 ± 0.10	0.56 ± 0.04	0.51 ± 0.13
fraction of protein remaining at the membrane after third rinse	n/a	0.49 ± 0.07	n/a
total amount of associated protein during protein incubation in units of volume per surface area** / Å	6.6 ± 1.1	7.0 ± 0.8	3.0 ± 1.5
distance of center of mass of protein from bilayer interface** / Å	23.6 ± 2.0	31.2 ± 1.6	25.2 ± 5.0
**Global Properties**			
goodness of best-fit	1.73	2.94	2.27

Table S7: **Neutron Reflection Model Fit Results.** Best-fit parameters and 68% confidence intervals using the Continuous Distribution model of a protein adsorbed to stBLMs, as derived from Monte Carlo resampling.

*nSLD of Cr bonding layers is found typically above the bulk value of 3.03 10^{-6} $Å^{-2}$ due to inter-diffusion of Cr and Au.

#In some cases a inter-diffusion of SiO_x and Permalloy is observed, which is best described by an elevated nSLD of the SiO_x.

**This value is computed from a combination of fit parameters.

References

1. Schasfoort R, Tudos A, eds. *Handbook of Surface Plasmon Resonance*. Cambridge: The Royal Society of Chemistry, 2008.

2. Vanderah DJ, Silin V, Weetall H. *SPR Studies of the Nonspecific Adsorption Kinetics of Human IgG and BSA on Gold Surfaces Modified by Self-Assembled Monolayers (SAMs)*. Journal of colloid and interface science. (1997) 185:94-103.

3. Liu Y, Daum P. *Relationship of Refractive Index to Mass Density and Self-Consistency of Mixing Rules for Multicomponent Mixtures Like Ambient Aerosols*. Aerosol Sci. (2008) 39:974–986.

4. Refractive Index Database. Available at: http://refractiveindex.info/?group= [Accessed January 2010].

5. Luxpop: *Thin Film and Bulk Index of Refraction and Photonics Calculations*. Available at: http://www.luxpop.com/ [Accessed January 2010].

6. De Feijter J, Benjamins J, Veer FA. *Ellipsometry as a Tool to Study the Adsorption Behavior of Synthetic and Biopolymers at the Air-Water Interface.* Biopolymers. (1978) 17:1759-1772.

7. Press W, Flannery B, Teukolsky S, Vetterling W, eds. (1986) *Numerical Recipes*. Cambridge, U.K.: Cambridge University Press.
